# Emerin modulates spatial organization of chromosome territories in cells on softer matrices

**DOI:** 10.1093/nar/gky288

**Published:** 2018-04-19

**Authors:** Roopali Pradhan, Devika Ranade, Kundan Sengupta

**Affiliations:** Biology, Main Building, First Floor, Room#B-216, Indian Institute of Science Education and Research (IISER), Pune, Dr Homi Bhabha Road, Pashan, Pune 411008, Maharashtra, India

## Abstract

Cells perceive and relay external mechanical forces into the nucleus through the nuclear envelope. Here we examined the effect of lowering substrate stiffness as a paradigm to address the impact of altered mechanical forces on nuclear structure-function relationships. RNA sequencing of cells on softer matrices revealed significant transcriptional imbalances, predominantly in chromatin associated processes and transcriptional deregulation of human Chromosome 1. Furthermore, 3-Dimensional fluorescence in situ hybridization (3D-FISH) analyses showed a significant mislocalization of Chromosome 1 and 19 Territories (CT) into the nuclear interior, consistent with their transcriptional deregulation. However, CT18 with relatively lower transcriptional dysregulation, also mislocalized into the nuclear interior. Furthermore, nuclear Lamins that regulate chromosome positioning, were mislocalized into the nuclear interior in response to lowered matrix stiffness. Notably, Lamin B2 overexpression retained CT18 near the nuclear periphery in cells on softer matrices. While, cells on softer matrices also activated emerin phosphorylation at a novel Tyr99 residue, the inhibition of which in a phospho-deficient mutant (emerinY99F), selectively retained chromosome 18 and 19 but not chromosome 1 territories at their conserved nuclear locations. Taken together, emerin functions as a key mechanosensor, that modulates the spatial organization of chromosome territories in the interphase nucleus.

## INTRODUCTION

The cytoskeleton perceives and relays altered extracellular forces into the nucleus in order to regulate growth, development and differentiation (1–4). The LINC (Linker of Nucleoskeleton and Cytoskeleton) complex communicates extracellular forces into the nucleus via cytoskeletal proteins on the cytoplasmic side and lamins at the inner nuclear membrane. Lamins transduce external mechanical signals into the genome to elicit appropriate mechanosensitive gene expression signatures and transcriptional responses (4–9). The nuclear lamina is a ‘molecular shock absorber’ that maintains nuclear morphology to counter extraneous mechanical tension, while lamin associated nuclear envelope proteins namely, emerin, LAP2β and MAN1 (LEM Domain proteins) regulate mechanotransduction into the nucleus (10–15). Interestingly, extracellular substrate stiffness modulates expression levels and phosphorylation of Lamin A (16–19). In addition, emerin is a mechanosensor that directly interacts with Lamin A/C and is phosphorylated in response to increased mechanical stress (20–22).

It is well established that the genome is non-randomly organized in the interphase nucleus, with gene rich chromosome territories toward the nuclear interior, while gene poor chromosome territories are proximal to the nuclear periphery (23–25). However, this otherwise conserved chromosome organization is altered during differentiation, senescence, quiescence, in serum starved cells or in cells treated with DNA damaging agents, within minutes to hours (26–32). Lamins interact with chromatin via Lamina-Associated Domains (LADs), tether heterochromatin to the nuclear periphery and modulate chromosome territory positions in the interphase nucleus (33,34). For instance, mouse chromosome 18 is shifted away from the nuclear periphery in Lamin B1 knockout murine cells (35). Loss of function or mutations in the LINC complex, the nuclear envelope proteins (like emerin) or the nuclear lamins leads to ‘Nuclear Envelopathies’ with aberrant nuclear morphologies and impaired mechanotransduction (8,22,36–39). Lamin A mutations in cardiomyopathies (E161K) and progeria (G608G) show aberrant chromosome positioning, gene expression profiles and epigenetic modifications (40–42). Furthermore, dermal fibroblast cell lines derived from laminopathy patients (R298L, E358K, R482L among others, with *LMNA* mutations) and X-EDMD patient derived dermal fibroblasts (ED5364, with *EMD* mutations) show mislocalization of gene poor chromosomes 13 and 18 away from the nuclear periphery (43). A mechanosensitive sub-complex of emerin, non-muscle myosin IIA and actin also tethers heterochromatin with the nuclear lamina (44). This underscores the importance of a structurally and functionally resilient nucleus in maintaining chromatin organization and function.

The impact of external mechanical forces on non-random chromosome positions and transcription is largely unclear. For instance, Hi-C studies reveal that chromatin organization differs significantly in human fibroblasts grown on 2D versus 3D microenvironments (45). Cells on micropatterned surfaces increase histone acetylation (AcH3) and methylation (H3K4me2/me3) levels, suggesting that altered substrate architecture is potentially perceived by the genome and fine-tuned by the epigenome (46–48). Micro-patterned surfaces alter Lamin B1 organization and mislocalize human chromosome 1 territories from a more central location towards the nuclear periphery (49). In addition, heterochromatinization and transcriptional repression is induced in cells on relatively softer matrices (<50 kPa), potentially relayed to the genome via the LINC complex (50–52). These studies reveal that changes in mechanical forces perceived by cells can impact chromosome organization and function.

Chromosome positions have been examined in cells cultured on tissue culture plastic or glass surfaces, whose stiffness is orders of magnitude higher (∼10 GPa) than that experienced by cells under physiological conditions (0.1–200 kPa), or in fixed non-adherent lymphocytes (24,53–56). Studies in murine and porcine tissues reveal tissue specific differences in the spatial positioning of chromosome territories, gene loci and expression of nuclear envelope transmembrane proteins (NETs) (57–60). These experiments suggest that *in vivo* tissue architecture and extracellular matrix stiffness can enforce cell type specific genome organization and gene expression programs.

Here, we show that exposing cells to lowered matrix stiffness significantly perturbs the transcriptome accompanied by a mislocalization of chromosome territories into the nuclear interior. Furthermore, cells on softer matrices induced emerin phosphorylation as well as the mislocalization of nuclear envelope proteins into the nucleoplasm. Remarkably, inhibiting emerin phosphorylation by mutating its Tyr99 residue (Y99F) abrogated the mislocalization of chromosome 18 and 19 territories, highlighting the role of emerin phosphorylation as a key mechanosensitive signal, which along with nuclear lamins modulates chromosome territory positions in the interphase nucleus.

## MATERIALS AND METHODS

### Cell culture

DLD-1 colorectal adenocarcinoma cells were obtained from the laboratory of Thomas Ried (NCI, NIH, Bethesda, USA). These cells were maintained in RPMI media (Invitrogen, RPMI 1640, 11875-093) supplemented with 10% fetal bovine serum (FBS, Invitrogen, 6140-079 Carlsbad, USA) and antibiotics—Penicillin (100 U/ml) and Streptomycin (100 μg/ml, Invitrogen, 15070-063) at 37°C with 5% CO_2_. DLD-1 cells were authenticated by karyotyping, which reconfirmed their near diploid modal chromosome number of 44–46 chromosomes (Supplementary Figure S1A). Cells in culture were routinely tested and found to be free of mycoplasma contamination.

### Preparation of metaphase spreads

DLD-1 cells were blocked in metaphase using 0.1 μg/ml Colcemid (Roche 10 295 892 001) for 90 min. Hypotonic treatment (using 0.075 M KCl) was performed for 30 min followed by fixation in 5–6 drops of fixative (Methanol:Acetic Acid, 3:1). After four washes with fixative, the cell suspension was dropped onto clean glass slides and metaphases were stained with DAPI.

### Preparation of polyacrylamide gels

Polyacrylamide gels were prepared following established protocols (61). Glass coverslips stored in 70% ethanol solution were sonicated in 1M KOH for 15 min in probe Sonicator (Sonics *VibraCell* Model No. VCX130; amplitude 50%, cycle 4 s ON/5 s OFF). After washing with Milli-Q water, coverslips were coated with 1% silane solution (3-aminopropyl-triethoxysilane, Sigma, 440140) for 40 min. Coverslips were cured at 50°C and allowed to dry completely, followed by treatment with 0.5% glutaraldehyde (Sigma, G7776) for 60 min. Polyacrylamide gels were prepared by the sandwich method using 5% acrylamide/0.2% bisacrylamide solution for softer (2 kPa) gel and 12% acrylamide/0.6% bisacrylamide solution for the stiffer (55 kPa) gel. Gels were activated using Sulpho-Sanpah (Pierce, 22589), followed by coating with 100 μg/ml rat-tail Collagen (BD Biosciences, 354236 and Sigma, C7661) at 4°C.

### Cell cycle analysis

DLD-1 cells plated on 2 kPa, 55 kPa matrices and collagen coated glass coverslips were subjected to cell cycle profiling using a fluorescence activated cell scanner (BD FACSCalibur™, BD Biosciences). Cells (∼0.8 million) were seeded on the matrices and glass coverslips for 90 min followed by trypsinization and centrifugation at 10°C/1000 rpm for 5 min. Cell pellets were washed once with DPBS and the pellets were resuspended in 1 ml 70% ethanol solution under constant agitation and stored at 4°C overnight. Cells fixed in 70% ethanol were centrifuged at 10°C/1050 rpm for ∼7–10 min. The pellet was resuspended in 1 ml 1× PBS followed by addition of 5–7 μl RNase A (stock: 10 mg/ml) and 10–12 μl propidium iodide (stock: 1 mg/ml) and incubated at 37°C for 60 min with intermittent tapping. Cell suspensions (2 kPa, 55 kPa and glass—stained, and glass—unstained control) were passed through a cell strainer and collected in FACS tubes. Cell cycle analysis was performed with ∼30 000 cell count per sample.

### Western blotting

Cell lysates were prepared using Radio Immuno-Precipitation Assay (RIPA) Buffer and quantified using BCA (Bicinchoninic Acid) Kit (Pierce, 23225). Samples were denatured by boiling in 4× Laemmli Buffer and resolved on either 10% or 15% acrylamide-bisacrylamide gel, followed by transfer to an activated PVDF membrane at constant voltage of 90 V for 100 min. The membrane was blocked in 5% non-fat dried milk prepared in 1× Tris Buffered Saline-Tween20 (1× TBST). Primary and secondary antibody dilutions were prepared in 0.5% milk in 1× TBST. Blots were developed using chemiluminescent substrate (GE ECL Prime, 89168-782) and images acquired at incremental exposures of 10 s under a chemiluminescence system LAS4000 (GE). Following molecular weight markers were used: Precision Plus Protein Dual Colour Standards (250-10 kDa, Biorad, Cat. No. 161-0374) and SeeBlue Prestained ladder (198-3 kDa, Invitrogen, P/N 100006636). Primary antibodies used were: Rabbit anti-Lamin A (ab26300, 1:1000), Rabbit anti-Lamin B1 (ab16048, 1:1000), Mouse anti-Lamin B2 (ab8983, 1:400), Rabbit anti-Lamin B2 (AV46356, 1:500), Rabbit anti-Emerin (06-1052, 1:3000), Rabbit anti-Emerin (ab40688, 1:1500), Rabbit anti-SUN1 (ab74758, 1:1000 and ab125770, 1:1000), Rabbit anti-SUN2 (ab124916, 1:1000), Rabbit anti-H3K4me3 (Millipore 07-473, 1:2000), Rabbit anti-H3K27me3 (Millipore 07-449, 1:2000), Rabbit anti-Histone H3 (ab1791, 1:2000), Mouse anti-Actin (ab3280, 1:400), Rabbit anti-GAPDH (G9545, 1:5000), Rat anti-Tubulin (ab6161, 1:6000) and Mouse anti-Phospho-tyrosine conjugated with HRP (610011, 1:1000). Secondary antibodies used were Sheep anti-mouse IgG-HRP (NA9310V, 1: 10 000), Donkey anti-rabbit IgG HRP (NA9340V, 1:10 000) and Goat anti-rat IgG-HRP (ab97057, 1: 10 000).

### Immunofluorescence assay

Cells plated on coverslips or polyacrylamide gels were washed twice using 1× PBS (5 min) followed by fixation with 4% Paraformaldehyde (PFA, Sigma, 158127) prepared in 1× PBS (pH 7.4). Cells were permeabilized in 0.5% Triton-X-100 (prepared in 1× PBS) and blocked in 1% BSA (Sigma, A2153) solution. Primary and secondary antibody incubations were carried out for 90 and 60 min respectively. Cells were counterstained with 0.05 μg/ml 4′,6-diamidino-2-phenylindole (DAPI) for 2 min at RT, washed in 1× PBS, mounted in Slowfade Gold Antifade (Invitrogen, S36937) and stored in 4°C until they were imaged. Following primary antibodies—Rabbit anti-Lamin A (ab26300, 1:500), Rabbit anti-Lamin B1 (ab16048, 1:500), Mouse anti-Lamin B2 (ab8983, 1:400), Mouse anti-Emerin (SC-25284, 1:500), Rabbit anti-SUN1 (ab125770, 1:500), Rabbit anti-SUN2 (ab124916, 1:500), Rabbit anti-H3K27me3 (07-449, 1:500) and Rabbit anti-H3K4me3 (07-473, 1:500) were used. Primary antibody dilutions were prepared in 0.5% BSA solution. Phalloidin conjugated to Alexa-488 (A12379, 1:100) and secondary antibodies—Goat anti-Rabbit Alexa-488 (A11034, 1:1000), Goat anti-Rabbit Alexa-633 (A21070, 1:750), Goat anti-Rabbit Alexa 568 (A11011, 1:1000) and Goat anti-Mouse Alexa-568 (A11004, 1:1000) were used. Secondary antibody dilutions were prepared in 1× PBST (1× PBS + 0.1% Triton X-100).

### Generation of Lamin A, Lamin B2 and Emerin mutants

The pEGFP-Lamin A and Lamin B2-GFP constructs were received as kind gifts from Kaushik Sengupta (SINP, Kolkata, India) and Takeshi Tomonaga (NIBIO, Osaka, Japan). GFP-Emerin and GFP-Emerin Δ95–99 constructs were received as kind gifts from Katherine Wilson (JHMI, Baltimore, USA). Lamin domain organization was obtained from Uniprot. Lamin A Δ425-553 and Lamin B2 Δ570-582 mutants were generated from full length constructs using the following primers—Lamin A Δ425-553 sense 5′-CAAACTGGAGTCCACTGAGGATGAGGATGGAG-3′, Lamin A Δ425-553 antisense 5′-CTCCATCCTCATCCTCAGTGGACTCCAGTTTG-3′, Lamin B2 Δ570-582 sense 5′-GGTTAACGCGGATGGCATGCGTGAGAATGAGA-3′ and Lamin B2 Δ570-582 antisense 5′- TCTCATTCTCACGCATGCCATCCGCGTTAACC-3′. Emerin Y74F, Y95F, Y74/95FF and Y99F single point mutants were made from GFP-Emerin plasmid using the following primers—Emerin Y74F sense 5′-TTCTTGGGAAGATCAAACATATCTGCATCCCCTCTAG-3′, Emerin Y74F antisense 5′-CTAGAGGGGATGCAGATATGTTTGATCTTCCCAAGAA-3′, Emerin Y95F sense 5′-GAAGTAGCTCTCTTCAAAGTAGTCGTCATTGTAGCC-3′, Emerin Y95F antisense 5′-GGCTACAATGACGACTACTTTGAAGAGAGCTACTTC-3′, Emerin Y99F sense 5′-AAGTCCTGGTGGTGAAGAAGCTCTCTTCATAGTAG-3′ and Emerin Y99F antisense 5′-CTACTATGAAGAGAGCTTCTTCACCACCAGGACTT-3′. WT GFP-Emerin and Emerin Y99F were rendered shRNA insensitive using the following primers—sense 5′-TGCACTCCTCTTCAGAAGAAGATAATAGGTCATCGTCGTGCACTTGGTGATGGAAAGCGTCAGCATCTG-3′ and antisense 5′-CAGATGCTGACGCTTTCCATCACCAAGTGCACGACGATGACCTATTATCTTCTTCTGAAGAGGAGTGCA-3′. Primers were generated using the QuikChange Primer Design software from Agilent Genomics. PCR was carried out using Accuprime *Pfx* Supermix (Invitrogen, 12344-040).

### Generation of emerin knockdown (shEmerin) clones of DLD-1 cells

DLD-1 cells (∼0.8 million) were seeded in a 60 mm culture dish, followed by transfection with either shRNA (8 μg) against emerin (pLKO.1/puro TRC1.5 vector backbone, Sigma TRCN0000083012) or pLKO.1 empty vector (as vector control) using Lipofectamine LTX and Plus reagent (Invitrogen, 15338100). Emerin shRNA sequence is as follows -5′CCGGAGGTGCATGATGACGATCTTTCTCGAGAAAGATCGTCATCATGCACCTTTTTTG3′. After 48 h, cells were trypsinized and seeded onto 100 mm culture dishes under Puromycin selection (2.5 μg/ml, Invitrogen, A1113802). The shEmerin colonies were screened using western blotting and immunofluorescence for emerin depletion (empty vector colonies were used as control). The selected vector control and shEmerin clones were maintained under continuous puromycin selection (2.5 μg/ml).

### Overexpression of Lamin A, Lamin B2 and Emerin

DLD-1 cells were transfected with overexpression vectors for Lamin A (GFP-Lamin A, GFP-Lamin A Δ425-553), Lamin B2 (Lamin B2-GFP, Lamin B2-GFP Δ570-582) and Emerin (GFP-Emerin WT, Y74F, Y95F, Y74/95FF, Y99F) using Lipofectamine LTX and Plus reagent (Invitrogen, 15338100) for 48 h. Following this, cells were trypsinized and plated on 2 kPa polyacrylamide gel (or glass—Lamin overexpression) for 90 min and then processed for western blotting (Lamin and Emerin overexpression) and 3D-FISH fixation (Lamin overexpression).

Overexpression of WT GFP-Emerin and GFP-Emerin Y99F (resistant to shRNA) in vector control and shEmerin clones was performed using TransIT-2020 transfection reagent (Mirus Bio LLC) at a concentration of 1.5 μl/μg of plasmid. Overexpression was carried out for 48 h, following which cells were trypsinized and plated on 2 kPa polyacrylamide gel for 90 min and then processed for western blotting, immunofluorescence or 3D-FISH fixation.

### 3-Dimensional fluorescence in situ hybridization (3D-FISH)

#### Fixation

Cells plated independently on glass coverslips (18 × 18 mm or 22 × 22 mm) and softer polyacrylamide matrices for 90 min were washed thrice in 1× PBS (5 min), incubated on ice for 6 min in CSK buffer (0.1 M NaCl, 0.3 M Sucrose, 3 mM MgCl_2_, 10 mM PIPES (pH 7.4), 0.5% Triton-X-100) and immediately fixed in 4% Paraformaldehyde (PFA, pH 7.4) for 15 min. Permeabilization was done in 0.5% Triton-X-100 for 15 min, incubation in 20% glycerol for 60 min, followed by 4–5 freeze-thaw cycles in liquid nitrogen. Cells were washed in 1× PBS (thrice/5 mins each), incubated in 0.1 N HCl for 10 min and washed in 1X PBS (thrice/5 mins each). Cells were stored in 50% formamide (FA)/2XSSC (pH 7.4) overnight at 4°C or until used for hybridization.

#### Hybridization

Chromosome painting probes were obtained from Applied Spectral Imaging (ASI, Israel) and MetaSystems (Germany). Probes were pre-warmed at 37°C for 5 min (with agitation at 750 rpm on a thermomixer) followed by denaturation at 80°C for 5 min (82°C for MetaSystems probes), and quick chilled on ice for 2 min followed by pre-annealing at 37°C for 45 min. Denatured probe (5 μl) was spotted onto fixed cells and subjected to co-denaturation at 80°C for 10 min (2 kPa matrix and glass coverslips) and 15 min (55 kPa matrix). Hybridization was carried out for 48 h in a humidified box at 37°C.

#### Detection

Post hybridization, coverslips were washed in 50% FA/2XSSC (pH 7.4), thrice/5 min each at 45°C, followed by 0.1× SSC washes (thrice/5 mins each) at 60°C. Coverslips were counterstained with DAPI for 2 min, washed in 2XSSC, mounted in Slowfade Gold Antifade and stored at 4°C until imaged.

#### Imaging

Confocal images were acquired on Zeiss LSM 710 confocal microscope (Carl Zeiss, Thornwood, NJ, USA) and Leica TCS SP8 confocal laser scanning microscope. LSM/LAS X image stacks were processed using Image Pro Plus software (v 7.1).

### Radial distance measurements of chromosome territories

3D reconstructions and radial distance measurements of chromosome territories were performed using Image-Pro Plus software (v 7.1). Briefly, LSM files with optical sections (*z* = 0.34 μm) of hybridized nuclei were subjected to 3D surface rendering. Individual nuclei were cropped for 3D reconstruction. The acquired images were thresholded for each of the red, green and blue channels. The geometric center of the DAPI stained nucleus (blue channel) and the chromosome territories (red and green channels) were determined using Image-Pro Plus software, and the distance between the centre of the nucleus and that of the territory was measured (*R*). The vector *R* from the centre of nucleus (*N*) to that of the chromosome territory (*C*) was extended to a third collinear point on the nuclear periphery (*B*). The distance (*Y*) between the centre of the nucleus and point B was calculated. The relative distance of a chromosome territory from the center of the nucleus was expressed as a percentage of its total distance from the center of the nucleus to the nuclear periphery, %radial distance = (*R/Y*) × 100 (56).

### Imaging and acquisition parameters

Confocal images were acquired on Zeiss LSM 710 confocal microscope (Carl Zeiss, Thornwood, NJ, USA) with 63× Plan-Apochromat 1.4 NA oil immersion objective using charge-coupled device camera (AxioCam MRm Rev.3, Zeiss), ZEN software and scan zoom of 2.0–2.5. *Z*-stacked images were acquired at 512 × 512 pixels per frame using 8-bit pixel depth for each channel at a voxel size of 0.105 μm × 0.105 μm × 0.34 μm and line averaging set to 2 collected sequentially in a three-channel mode. Imaging was also performed using Leica TCS SP8 confocal laser scanning microscope with 63× Plan-Apochromat 1.4 NA oil immersion objective, LAS X software and scan zoom of 1.5–2.0. *Z*-stacked images were acquired at 512 × 512 pixels per frame using 8-bit pixel depth for each channel at a voxel size of 0.105 μm × 0.105 μm × 0.34 μm and frame averaging set to four collected sequentially in a three-channel mode. Slides were mounted in Slowfade Gold Antifade and fluorochromes used were as follows: DAPI, Alexa Fluor-488 and Alexa Fluor-568. LSM/LAS X image stacks were processed using Image Pro Plus software (v 7.1). High resolution imaging of Emerin GFP-tagged constructs was performed using Leica TCS STED 3X Nanoscope using 100X HC Plan-Apochromat 1.4 NA oil immersion objective. Depletion lasers of 592 nm and 660 nm were used for GFP and Alexa Fluor 568 respectively. Acquisition was carried out using the LASX software, scan zoom between 4–6 and 1352 × 1352 pixels per frame at a voxel size of 0.03 μm × 0.03 μm × 1 μm.

### RNA sequencing

DLD-1 cells were plated on 2 kPa, 55 kPa polyacrylamide matrices and collagen coated glass coverslips for 90 min. Cells were harvested in Trizol^®^ reagent. RNA-Sequencing over two independent biological replicates was performed by Genotypic Technology, Bangalore on Illumina NextSeq 500 sequencing platform followed by quality control of paired end raw reads using FastQC v2.2. Reference genome alignment was performed using TopHat v2.0.7 and Cufflinks v2.0.1, and differential expression analysis was performed using Cufflinks v2.0.1—Cuffdiff (62–64). Homology searches were performed against Ensemble cDNA sequences (GRch37/hg19 build) using ncbi-BLAST-2.2.29. Spearman and Pearson correlation coefficients between the two independent biological replicates (for the soft matrices) was performed using deepTools2 as a part of the Galaxy platform (65,66). The total deregulation (%) on each chromosome was calculated by normalizing total number of deregulated genes on each chromosome (on both 2 kPa and 55 kPa, reference—glass) to the total number of transcribing genes (FPKM > 1.0) on that chromosome. Enrichment of deregulated genes (up- and downregulated) on each chromosome on 2 kPa and 55 kPa matrices was calculated by normalizing total number of deregulated genes on each chromosome to the total number of genes deregulated on the soft (2 or 55 kPa) matrices (reference—glass). GO categories for up- and downregulated genes were assigned using DAVID Bioinformatics Resource 6.7 (NIAID, NIH). Briefly, genes up and downregulated [fold change ≥ 2-fold (log_2_)] on either 2 kPa (reference—glass) or 55 kPa (reference—glass) matrices were analyzed using the Functional Annotation Tool. Categories with *P* < 0.05 were plotted as the –log_10_(*P* value).

### Statistical analysis

The frequency distribution of %radial distance (%RD) was plotted in bins of 20% RD. The radial distances of chromosome territories were compared between independent categories using the Mann–Whitney–Wilcoxon sum rank test using Graph Pad Prism 5.0 software. The densitometric (western blotting) and fluorescence intensity (IFA) values were compared using Student's *t*-test, *P*-value < 0.05 was considered statistically significant. Graphs were plotted using Graph Pad Prism 5.0 and Microsoft Excel.

## RESULTS

### Increase in cell and nuclear surface area on softer matrices plateau by ∼90 minutes

Cells experience a wide range of stiffness based on their tissue microenvironment. For instance, brain tissue is softer (∼0.2 kPa), while bone is considerably stiffer (50–200 kPa) (67–70). We measured cell and nuclear surface areas of diploid DLD-1 cells exposed to softer polyacrylamide matrices (2 kPa and 55 kPa), for increasing durations (Supplementary Figure S1A and B). We selected a stiffness of ∼2 and ∼55 kPa, as this mimics the elastic modulus of tissues such as the colon, intestine (∼2.9 kPa) and bone (>40 kPa) (69–72). We exposed cells to softer matrices from ∼15 min up to ∼21 h (Supplementary Figure S1B). Cell and nuclear surface area increased with time but plateaued at ∼90 min (Supplementary Figure S1C–F, Supplementary Table S1). We therefore selected a duration of 90 min for all our assays. Of note, we did not detect any significant sub-populations of arrested or senescent DLD-1 cells at the end of ∼90 min, as assessed by Fluorescence assisted cell scanning (FACS) analyses (Supplementary Figure S1G–I).

### Transcriptional deregulation in cells on softer matrices

**Figure 1.**
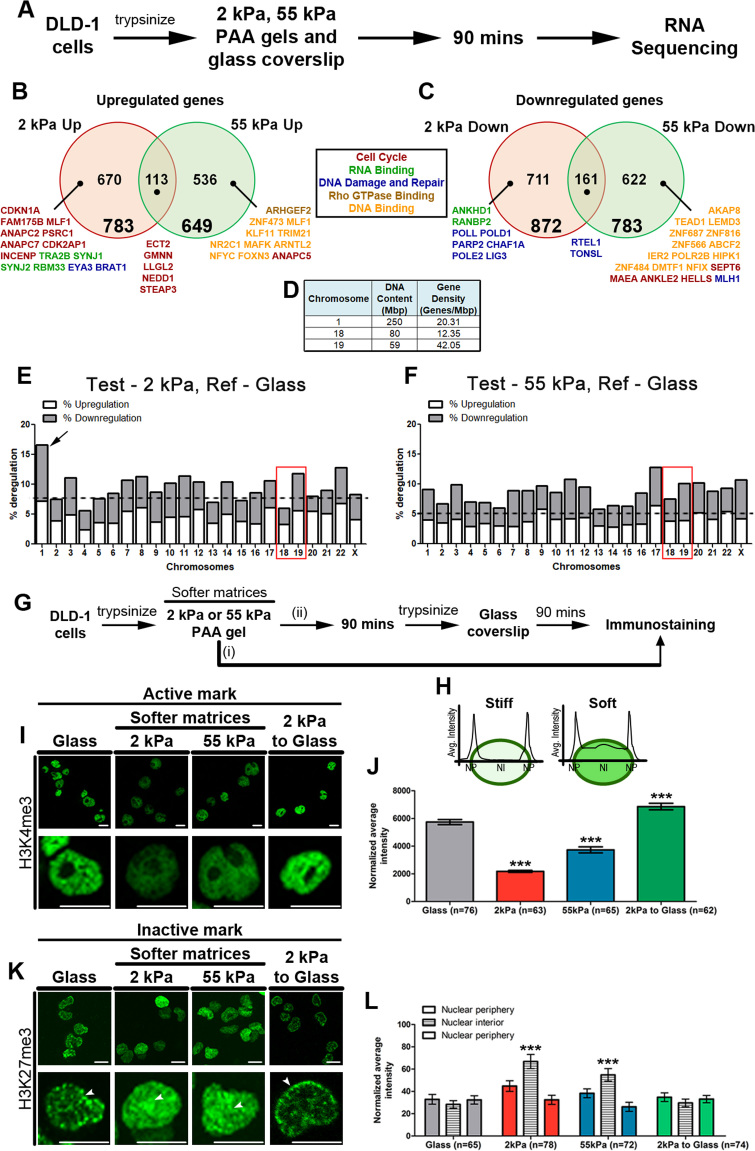
Transcriptional deregulation and mislocalization of inactive histone marks is induced in cells exposed to softer matrices. (**A**) Experimental scheme. RNA sequencing was performed in two independent biological replicates (Pearson correlation coefficient: 2 kPa replicates—0.94, 55 kPa replicates —1.0, Spearman correlation coefficient: 2 kPa replicates—0.90, 55 kPa replicates—0.90). (**B**) Total number of genes upregulated (≥log_2_- 2 fold) on the softer matrices—2 kPa (783 genes) and 55 kPa (649 genes), 670 and 536 genes were uniquely upregulated on 2 kPa and 55 kPa matrices respectively, while 113 genes were commonly upregulated on both the matrices. Selected genes from the maximally deregulated GO categories that are upregulated uniquely (>log_2_- 10 fold) and commonly (>log_2_ -2 fold) on both the soft matrices are displayed. (**C**) Total number of genes downregulated (≥log_2_- 2 fold) on the softer matrices—2 kPa (872 genes) and 55 kPa (783 genes), 711 and 622 genes were uniquely downregulated on 2 kPa and 55 kPa matrices respectively, while 161 genes were commonly downregulated on both the matrices. Selected genes from the maximally deregulated GO categories that are downregulated uniquely (>log_2_ - 10 fold) and commonly (>log_2_- 2 fold) on both the soft matrices are displayed. (**D**) Table depicting DNA content and Gene density of Chr. 1, 18 and 19. (**E**) Stacked bar graph depicting % deregulation (up and down) in cells on 2 kPa matrices, on all the chromosomes. Total number of ≥ log_2_ - 2 fold deregulated genes on each chromosome were normalized to the total number of transcribing genes (FPKM > 1) on that chromosome. *(Arrow)* Chromosome 1 shows the maximum deregulation on 2 kPa (∼16.64%). *(Red box)* Chromosome 18 is amongst the chromosomes showing least transcriptional changes, while chromosome 19 is amongst the chromosomes showing high transcriptional deregulation. (**F**) Stacked bar graph depicting % deregulation (up and down) in cells on 55 kPa matrices, on all the chromosomes. Total number of ≥log_2_ - 2 fold deregulated genes on each chromosome were normalized to the total number of transcribing genes (FPKM > 1) on that chromosome. *(Red box)* Chromosome 18 shows less transcriptional deregulation as compared to chromosome 19. (**G**) Experimental scheme. (**H**) Representation of fluorescence intensity quantification for each nucleus using line-scan analysis. (**I** and **J**) Representative mid-optical sections from confocal *z*-stack of DLD-1 cells immunostained for H3K4me3 on softer matrices (2 kPa and 55 kPa), glass coverslips and cells switched from 2 kPa to glass. Lower panel: zoom of single nucleus (J) Normalized average total fluorescence intensity of H3K4me3 under the above conditions (normalized to total nuclear surface area). (**K** and **L**) Representative mid-optical sections from confocal z-stack of DLD-1 cells immunostained for H3K27me3 on softer matrices (2 kPa and 55 kPa), glass coverslips and cells switched from 2 kPa to glass. Lower panel: zoom of single nucleus (L) Normalized average fluorescence intensity from line-scans across nuclei of H3K27me3 under the above conditions (J and L: *n*: number of nuclei, Pooled data from *N* = 2 independent biological replicates, Error bar: SEM, Mann–Whitney test). ***P < 0.0001. Scale bar ∼10 μm.

It is well established that the extracellular matrix modulates gene expression programs (52,73–76). We determined the effect of lowering matrix stiffness on the cellular transcriptome by performing RNA-Seq analyses of diploid DLD-1 cells exposed to softer matrices for 90 min (Figure 1A). Cells on collagen coated glass coverslips for the same duration served as reference (Figure 1A). RNA-Seq analyses revealed 783 genes that were upregulated, while 872 genes were downregulated in cells on the 2 kPa matrices (log fold ≥ 2) (Figure 1B and C). In contrast, 649 genes were upregulated and 783 genes were downregulated in cells on the 55 kPa matrices (log fold ≥ 2) (Figure 1B and C). We further classified up and downregulated genes into bins of increasing fold change, in order to identify chromosomes that were transcriptionally deregulated (Supplementary Figure S2A-D). Most chromosomes showed an equivalent extent of up and downregulation on both the matrices (Supplementary Figure S2C–F, Supplementary Tables S2 and S3). Interestingly, Chr.1 showed maximum transcriptional deregulation (∼16.64%) in cells on the 2 kPa matrices, while all other chromosomes were transcriptionally deregulated to an average of ∼9% (Figure 1D and E, Supplementary Figure S2E, Supplementary Table S2). We next examined human Chr. 18 and 19, as they represent chromosomes of divergent gene densities but comparable DNA content (Figure 1D). Gene rich Chr. 19 was transcriptionally deregulated to a greater extent than gene poor Chr. 18 in cells on either of the matrices (2 or 55 kPa) (Figure 1E and F, red box, Supplementary Figure S2C–F, red box, Supplementary Tables S2 and S3).

We performed Gene Ontology (GO) analyses using DAVID to identify the categories of significantly deregulated genes in cells exposed to softer matrices (77,78) (Supplementary Figures S3 and S4). This analysis revealed distinct subsets of genes associated with (i) mRNA processing, splicing and export (RNA binding category) (ii) cell cycle and (iii) DNA damage and repair that were strikingly up and downregulated on 2 kPa matrices (Supplementary Figure S3A and B). Genes associated with the Rho-GTPase signaling pathway were significantly upregulated and those associated with (i) transcription regulation, chromatin and chromosome organization (DNA binding category) (ii) cell cycle and (iii) DNA damage and repair were downregulated in cells on the 55 kPa matrices (Supplementary Figure S4A and B). These processes crosstalk with chromatin and influence its organization, which may be further modulated by the stiffness of the extracellular substrate (13,79). Of note, although common pathways were both up and downregulated on either of the matrices, non-overlapping and unique subsets of genes were deregulated in each of these categories. It is noteworthy that the upregulation of genes associated with the Rho-GTPase pathway i.e FMNL3, ARHGEF2, ARHGEF16, AKAP13, IQGAP2, MYO9B, ECT2 and DOCK11 on the 55 kPa matrices is consistent with their role in modulating cytoskeletal organization through Rho proteins, Rac1 and Cdc42 among others. This suggests the involvement of these genes in substrate stiffness-dependent mechanotransduction, generation of traction and induction of migration of these cells (80–87) (Supplementary Figure S4A).

### Inactive histone marks are mislocalized into the nuclear interior in cells on softer matrices

The levels and distribution of histone marks modulate gene expression (88). Histone marks such as H3/H4 lysine acetylation, H3K4me3, H3K79me3 and H3K36me3 are generally associated with transcriptional activation (89). While inactive histone marks such as H3K9me2/3 and H3K27me3 are predominantly associated with transcriptional repression, and are typically enriched as foci associated with heterochromatin predominantly at the nuclear periphery (89–91). Since gene expression levels were deregulated in cells on softer matrices (Figure 1B and C, Supplementary Figure S2C–F), we examined the nuclear localization and expression of active and inactive histone marks by immunofluorescence staining followed by confocal imaging, and independently by western blotting (Figure 1G–L, Supplementary Figure S2G–J). Interestingly, quantification of the fluorescence intensities of the active mark (H3K4me3) from immunofluorescence staining or overall levels from western blots showed a significant reduction (∼3-fold on 2 kPa and ∼1.5-fold on 55 kPa) in cells on softer matrices after ∼90 min (Figure 1I–J, Supplementary Figure S2G–H). These levels increased in cells transferred from the softer matrices to the stiffer glass substrates (Figure 1I–J, Supplementary Figure S2G and I). In contrast, the inactive mark (H3K27me3), otherwise enriched toward the nuclear periphery, mislocalized to the nuclear interior in cells on softer matrices (Figure 1K–L). Furthermore, the inactive mark (H3K27me3 foci) was restored to the nuclear periphery in cells transferred from the softer to stiffer matrices (glass) in ∼90 min (Figure 1K–L). However, there was no change in the overall levels of the inactive mark in cells on softer matrices (Supplementary Figure S2G–J). In summary, cells on softer matrices show (i) a decrease in the overall levels of active marks and (ii) mislocalization of inactive histone marks into the nuclear interior, consistent with the destabilization of the cellular transcriptome and suggestive of a relatively more repressed genomic configuration.

### Chromosome territories are mislocalized into the nuclear interior in cells on softer matrices

**Figure 2.**
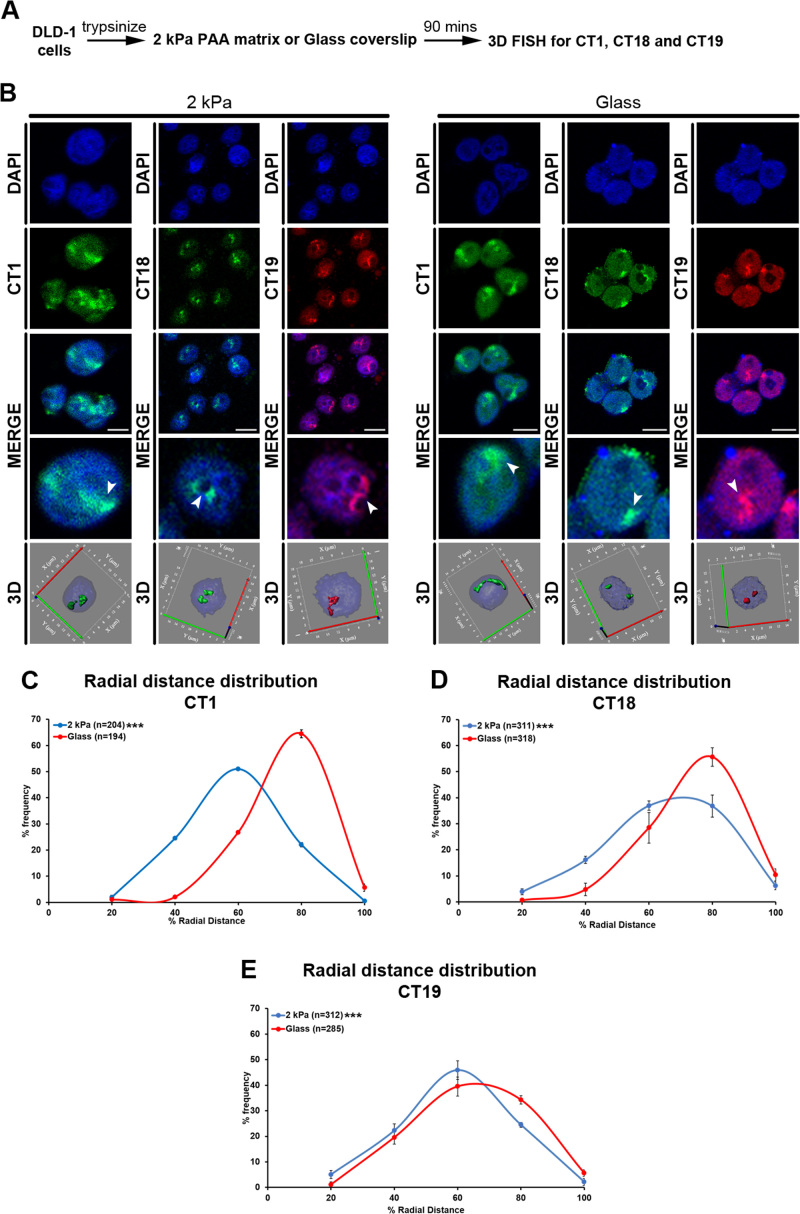
Chromosome territories are mislocalized into the nuclear interior in cells on softer matrices. (**A**) Experimental scheme. (**B**) Representative mid-optical sections from 3D-FISH hybridization for CT1, CT18 and CT19 in DLD-1 cells on softer matrices (2 kPa) and glass for 90 mins. Arrowheads show specific hybridization for CT1 (green), CT18 (green) and CT19 (red), resolved in 3D: reconstruction of single representative nucleus. (**C**) Radial distance distribution profiles for CT1 on 2 kPa (*N = 2, M* = 50.57%) and glass (*N* = 2, *M* = 66.81%) for 90 min. (**D**) Radial distance distribution profiles for CT18 on 2 kPa (*N = 3, M* = 56.56%) and glass (*N = 3, M* = 66.38%) for 90 min. (**E**) Radial distance distribution profiles for CT19 on 2 kPa (*N = 3, M* = 49.40%) and glass (*N* = 3, *M* = 54.73%) for 90 min (C–E: Pooled data from *N* independent biological replicates, *n*: number of CTs, X-axis: 0%—nuclear center and 100%—nuclear periphery, Error bar: SEM, Mann–Whitney test). ***P < 0.0001. Scale bar ∼10 μm.

We sought to examine the spatial organization of chromosome territories in cells on softer matrices (Figure 2), for which we quantified the radial distances of Chr. 1, 18 and 19 territories in the interphase nucleus as (i) Chr. 1 was maximally deregulated in cells on the softer (2 kPa) matrices (Figure 1E, Supplementary Figure S2C–F) (ii) gene poor Chr. 18 and gene rich Chr. 19 represent chromosomes of strikingly divergent gene densities but of comparable DNA content respectively (Figure 1D). Cells exposed to collagen coated glass coverslips for ∼90 min served as control (referred to as glass hereafter) (Figure 2A). Remarkably, 3D fluorescence in situ hybridization (3D-FISH) followed by confocal imaging and radial distance measurements of chromosome territories (56), showed that CT1 was strikingly mislocalized toward the nuclear interior (R.D ∼50.57%) in cells on softer matrices (2 kPa), from its otherwise conserved location closer to the nuclear periphery in cells on glass (R.D ∼66.81%) (Figure 2B–C, Supplementary Figure S5C, Table 2, Supplementary Table S5).

**Figure 3.**
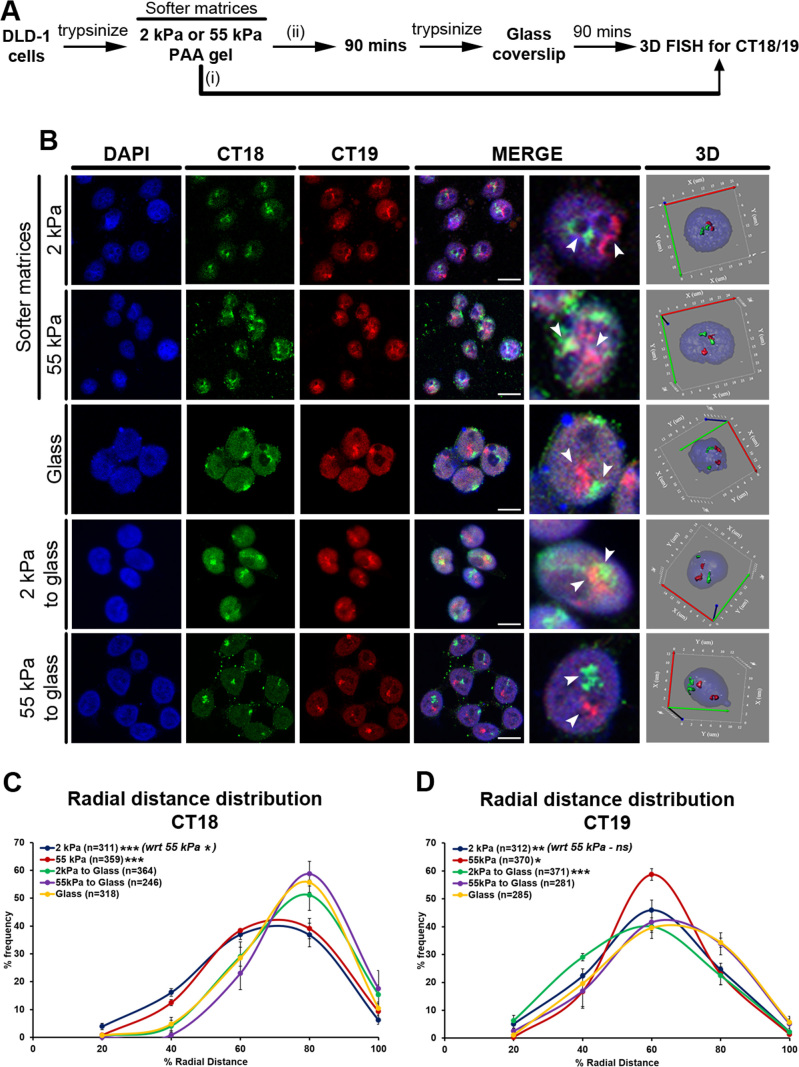
Chromosome 18 territories are restored to their conserved positions in cells transferred from softer to stiffer matrices. (**A**) Experimental scheme. (**B**) Representative mid-optical sections from 3D-FISH hybridization for CT18 and CT19 in DLD-1 cells on softer matrices (2 kPa—from Figure 2, and 55 kPa) and glass (from Figure 2) for 90 min, and in cells switched from softer matrices to glass. Arrowheads show specific hybridization for CT18 (green) and CT19 (red), resolved in 3D: reconstruction of single representative nucleus. (**C**) Radial distance distribution profiles for CT18 on 2 kPa (*N* = 3, *M* = 56.56%), 55 kPa (*N* = 3, *M* = 59.72%) matrices and glass (*N* = 3, *M* = 66.38%) for 90 mins, and in cells switched back to glass (*N* = 3, 2 kPa to glass: *M* = 67.11%, 55 kPa to glass: *M* = 68.75%). (**D**) Radial distance distribution profiles for CT19 on 2 kPa (*N* = 3, M = 49.40%), 55 kPa (*N* = 3, M = 50.01%) matrices and glass (*N* = 3, M = 54.73%) for 90 mins, and in cells switched back to glass (*N* = 3, 2 kPa to glass: *M* = 47.04%, 55 kPa to glass: *M* = 52.86%) (C–D: Pooled data from *N* independent biological replicates, *n*: number of CTs, X-axis: 0%—nuclear center and 100%—nuclear periphery, error bar: SEM, Mann–Whitney test). ***P < 0.0001, **P < 0.001, *P < 0.05. Scale bar ∼10 μm.

It is well established that the spatial positions of human Chr. 18 (gene poor, peripheral) and 19 (gene rich, internal) territories in the nucleus are non-random and are largely conserved across cell types (24,25,56). 3D-FISH, confocal imaging and radial distance measurements (R.D) of CT18 and CT19 in cells on glass consistently recapitulated their relatively peripheral and interior nuclear locations respectively (CT18: R.D ∼66.38%, CT19: R.D ∼54.73%; Figure 2B and D–E, Supplementary Figure S5A–B, Table 1, Supplementary Table S4). Remarkably, the gene poor CT18 significantly mislocalized toward the nuclear interior in cells on softer matrices (2 kPa: R.D ∼56.56%, 55 kPa: R.D ∼59.72%) from its otherwise peripheral nuclear localization in cells on glass (R.D ∼66.38%) (Figures 2D and 3B–C, Supplementary Figure S5A, Table 1, Supplementary Table S4). Furthermore, CT19 also shifted more into the nuclear interior in cells on softer matrices (2 kPa: R.D ∼49.40%, 55 kPa: R.D ∼50.01%, Glass: R.D ∼54.73%) (Figures 2E and 3B and D, Supplementary Figure S5B, Table 1, Supplementary Table S4). Interestingly, CT18 and C19 retained their mislocalized state even upon prolonged exposure to the softer matrices for ∼7 and ∼21 h respectively (7 h: CT18 R.D ∼55.91%, CT19 R.D ∼45.30%; 21 h: CT18 R.D ∼56.04%, CT19 R.D ∼45.42%; Supplementary Figure S5D–I). Taken together these results reveal that the spatial positions of chromosome territories are sensitive to reduced matrix stiffness and are consequently mislocalized toward the nuclear interior.

**Table 1.** Radial distance measurements of CT18 and CT19 under conditions of altered matrix stiffness

	Median % radial distance (% RD)				
Substrate/conditions	CT18	Δ	CT 19	Δ	Δ (CT18 – CT19)
(I) CT positions on softer matrices after 90 min (reference for comparison: glass)					
2 kPa (90 min)	**56.56 (*P* < 0.0001)**	0	**49.40 (*P* = 0.0007)**	0	7.16
55 kPa (90 min)	**59.72 (*P* < 0.0001)**	+3.16	**50.01 (*P* = 0.0126)**	+0.61	9.71
Glass (90 min)	66.38	+9.82	54.73	+5.33	11.65
2 kPa to glass	67.11	+10.55	**47.04 (*P* < 0.0001)**	−2.00	20.07
55 kPa to glass	68.75	+12.19	52.86	+3.46	15.89
(II) CT positions upon Lamin overexpression on softer matrices (2 kPa) (reference for comparison: EGFP-N1)					
EGFP-N1 on 2 kPa	56.79	+0.23	50.98	+1.58	5.81
GFP-Lamin A on 2 kPa	**46.41 (*P* < 0.0001)**	−10.15	**58.46 (*P* < 0.0001)**	+9.06	12.05
Lamin B2-GFP on 2 kPa	**67.41 (*P* < 0.0001)**	+10.85	**53.67 (*P* = 0.0054)**	+4.27	13.74
GFP-Lamin A Δ425-553 on 2 kPa	57.08	+0.52	48.73	−0.67	8.35
Lamin B2-GFP Δ570-582 on 2 kPa	54.07	−2.49	48.93	−0.47	5.14
(III) CT positions upon PP2 treatment on softer matrices (2 kPa) (Reference for comparison: respective DMSO control)					
Glass + DMSO	66.31	+9.75	53.15	+3.75	13.16
Glass + 20 μM PP2	67.93	+11.37	54.56	+5.16	13.37
2 kPa + DMSO	54.28	−2.28	48.93	−0.47	5.35
2 kPa + 20 μM PP2	**66.83 (*P* < 0.0001)**	+10.27	**53.83 (*P* < 0.0001)**	+ 4.43	13.00
(IV) CT positions upon Emerin Y99F overexpression on softer matrices (2 kPa) (Reference for comparison: Vector control+EGFP-N1)					
Vector control + EGFP-N1 on 2 kPa	51.43	−5.13	42.74	−6.66	8.69
Vector control + WT-EMD on 2 kPa	54.59	−1.97	43.69	−5.71	10.9
Vector control + EMD Y99F on 2 kPa	56.22	−0.34	47.49	−1.91	8.73
shEmerin + EGFP-N1 on 2 kPa	**61.80 (*P* < 0.0001)**	+5.24	**51.13 (*P* < 0.0001)**	+1.73	10.67
shEmerin + WT-EMD on 2 kPa	55.72	−0.84	47.50	−1.9	8.22
shEmerin + EMD Y99F on 2 kPa	**65.25 (*P* < 0.0001)**	+ 8.69	**53.24 (*P* < 0.0001)**	+3.84	12.01

Median radial distances of CT18 and CT19. Δ: shift in CT position, calculated with 2 kPa as reference, ‘+’: movement towards the nuclear periphery, ‘–’: movement towards the nuclear center. Δ (CT18-CT19): shift in CT position between CT18 and CT19. Values in bold are significant (*P* value in brackets).

### Chromosome 18 territories regain their conserved positions in cells transferred from softer to stiffer matrices

As chromosome territory positions are remarkably sensitive in cells on a softer milieu (Figure 2C–E), we asked if chromosome 18 and 19 territories with comparable DNA content but of contrasting gene densities, are responsive in cells transferred back to stiffer substrates (Figure 3A–B). Remarkably, the gene poor CT18 relocalized to its conserved position closer to the nuclear periphery within ∼90 minutes in cells transferred from either of the matrices to glass (2 kPa to glass: R.D ∼67.11%, 55 kPa to glass: R.D ∼68.75%) (Figure 3C, Supplementary Figure S5A, Table 1, Supplementary Table S4). In contrast, the gene rich CT19 remained relatively unperturbed near the nuclear interior in cells transferred from the softer matrices (2 kPa) to the significantly stiffer glass substrates, but shifted marginally away from the nuclear interior in cells switched from the 55 kPa matrices to glass (2 kPa to glass: R.D ∼47.04%, 55 kPa to glass: R.D ∼52.86%) (Figure 3D, Supplementary Figure S5B, Table 1, Supplementary Table S4). Furthermore, CT18 also re-positioned toward the nuclear periphery in cells transferred between the two substrates i.e from 2 kPa to 55 kPa, while positions of gene rich CT19 remained relatively unaltered (Supplementary Figure S6A–F). In summary, gene poor CT18 responds and repositions when transferred to stiffer matrices, while the gene rich CT19 near the nuclear interior is relatively less sensitive to an increase in matrix stiffness. Taken together, chromosome territory positions respond differentially in cells exposed to extracellular matrices of altered stiffness properties.

### Emerin phosphorylation is induced in cells on softer matrices

**Figure 4.**
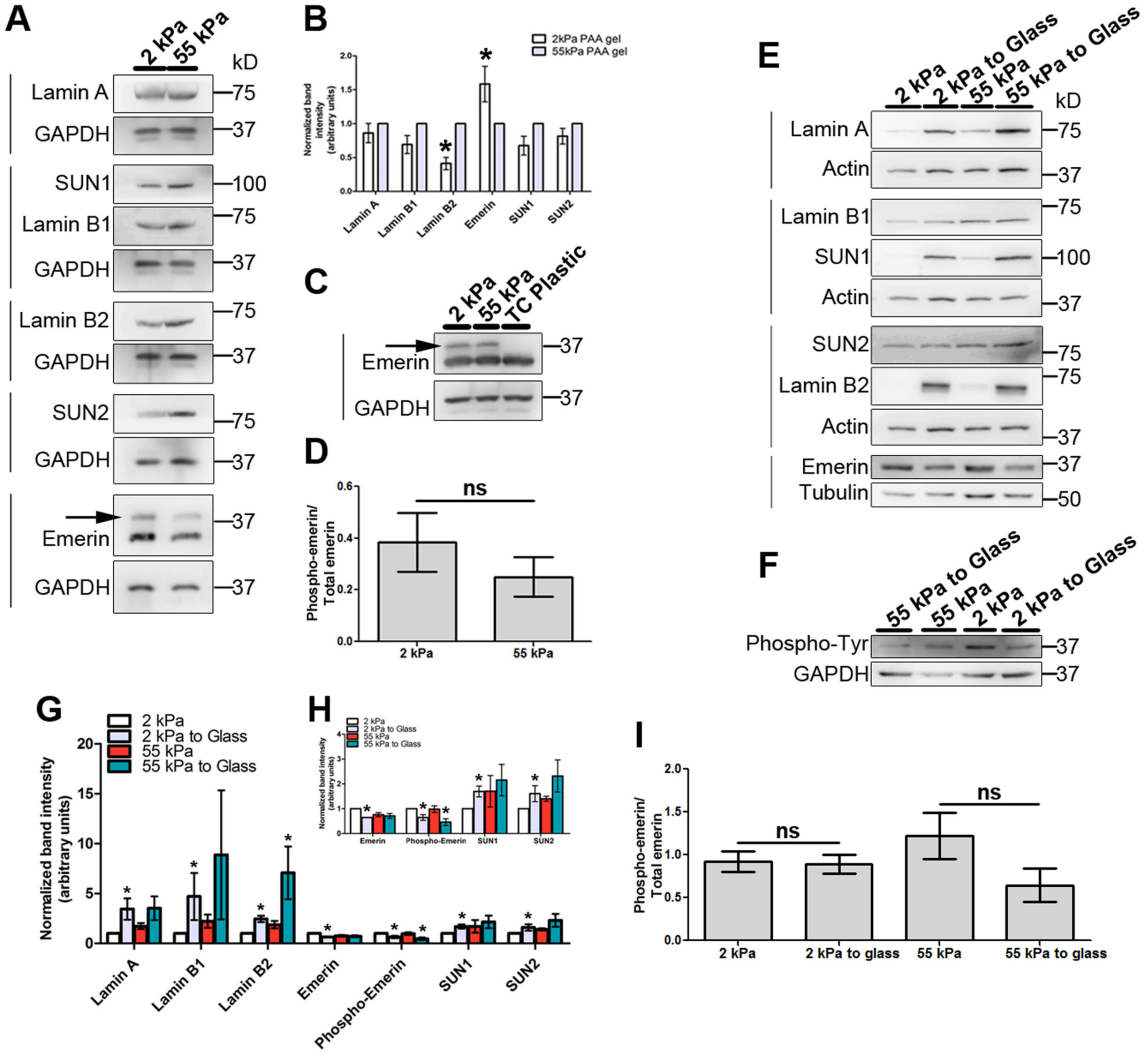
Reduced levels of nuclear envelope factors and induction of Emerin phosphorylation in cells on softer matrices. (**A**) Representative western blots (*N* = 6) for Lamin A, SUN1, Lamin B1, Lamin B2, SUN2 and emerin expression levels in DLD-1 cells on softer matrices (2 kPa and 55 kPa) after 90 min. Loading control: GAPDH. Arrow indicates phosphorylated emerin. (**B**) Densitometric quantification of expression levels of Lamins, emerin and SUN proteins on softer matrices. Expression was normalized to GAPDH and re-normalized to 55 kPa (error bars: SEM, pooled data from *N* = 6, Student's *t*-test). *P < 0.05. (**C**) Representative immunoblot (*N* = 3) showing emerin expression levels on softer matrices (2 kPa and 55 kPa) and tissue culture (TC) plastic. Loading control: GAPDH. Arrow indicates phosphorylated form of emerin on softer matrices. (**D**) Graph depicting phospho-emerin/emerin ratio for DLD-1 cells on softer matrices calculated from densitometric quantification of blots from Figure 4A (*N* = 6, error bar: SEM, Student's *t*-test). (**E**) Representative western blots (*N* = 3) for Lamin A, Lamin B1, SUN1, SUN2, Lamin B2 and emerin expression upon switching cells from softer matrices to glass. Loading controls: Actin and Tubulin. (**F**) Representative western blot (*N* = 3) for phospho-tyrosine expression upon switching cells from softer matrices to glass. Loading control: GAPDH. (**G**–**H**) Densitometric quantification of expression levels of Lamins, Emerin and SUN proteins from western blots in (E–F). Expression levels were normalized to loading control and re-normalized to 2 kPa (*N* = 3, error bars: SEM, Student's *t*-test). *P < 0.05. (**I**) Graph depicting phospho-emerin/emerin ratio for DLD-1 cells on softer matrices and switched from softer matrices to glass, calculated from densitometric quantification of blots from Figure 4E–F (*N* = 3, error bar: SEM, Student's *t*-test, For A-I, *N*: independent biological replicates).

To elucidate the mechanisms that modulate chromosome positioning in cells exposed to reduced matrix stiffness, we examined the levels of proteins that maintain and regulate nuclear architecture (41,92–94). Immunoblotting showed a marginal decrease in the levels of Lamin A, Lamin B1, SUN1 and SUN2, and a significant decrease in Lamin B2 levels in cells on softer matrices (2 kPa) within ∼90 min (Figure 4A–B). In sharp contrast, emerin levels increased (Figure 4A–B), with the prominent activation of emerin phosphorylation on both the matrices (Figure 4C, Supplementary Figure S6G). The overall levels of phosphorylated emerin were comparable on both the softer matrices (2 kPa and 55 kPa) as revealed by immunoblotting (Figure 4D). We hardly detected emerin phosphorylation in cells plated on the stiffer plastic substrates for 90 minutes (Figure 4C, compare lanes: 2 kPa, 55 kPa with TC plastic). Furthermore, emerin phosphorylation on softer matrices sustained up to ∼7 h, but decreased by ∼21 h (Supplementary Figure S6H). Notably, Lamin A, SUN1 and Lamin B2 levels increased and were restored in cells transferred from the softer matrices to the stiffer glass substrates (Figure 4E, G and H). However, emerin phosphorylation was retained even after cells were transferred to the stiffer glass substrates (Figure 4F and I). Taken together, cells show a distinctive reduction in the levels of nuclear envelope factors, but a striking activation and increase in emerin phosphorylation. This suggests the involvement of nuclear envelope factors as responders and effectors of the signaling cascade that perceive and relay stiffness properties of the extracellular substrate into the nucleus.

### Lamin/LINC factors are mislocalized into the nuclear interior in cells on softer matrices

**Figure 5.**
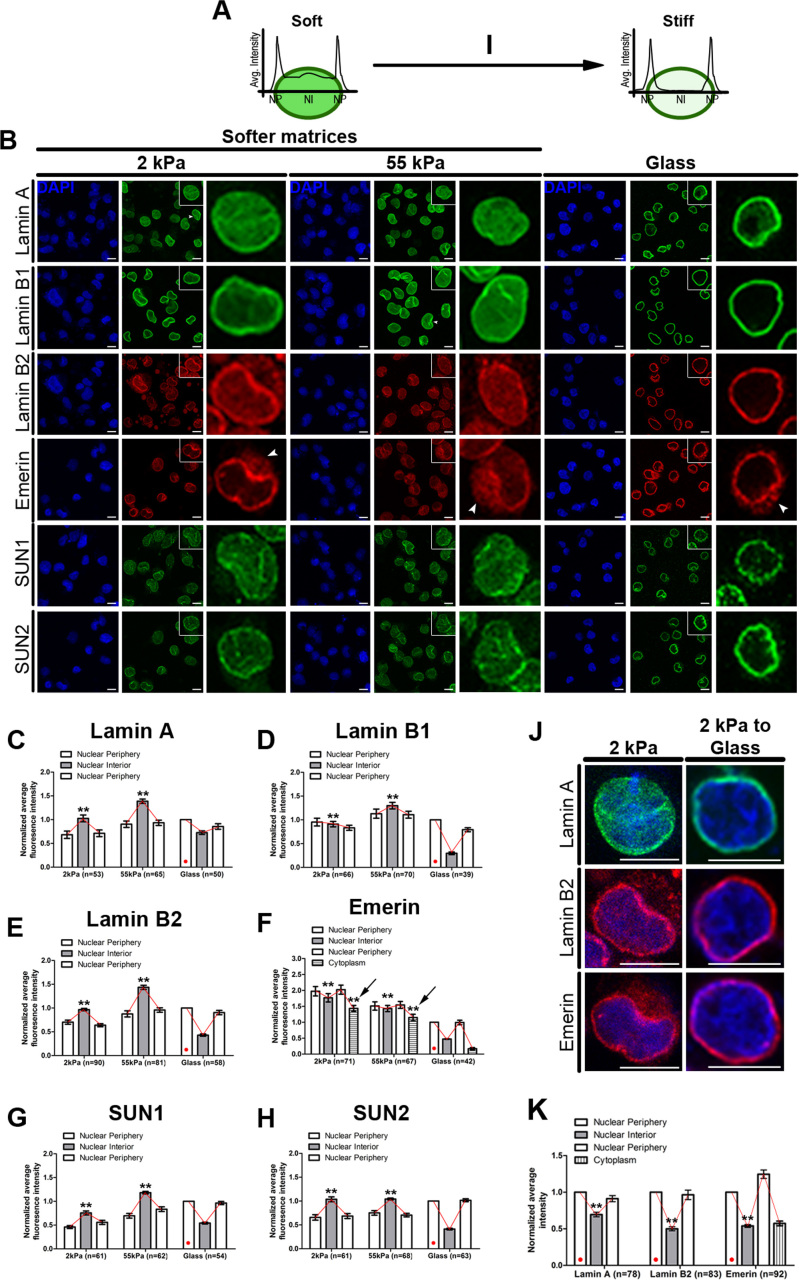
Lamin/LINC factors are mislocalized into the nuclear interior in cells on softer matrices. (**A, I**) Scheme represents fluorescence intensity quantification for each nucleus using line-scan analysis. (**B**) Representative mid-optical sections (*N* = 2 independent biological replicates) of DLD-1 cells immunostained for Lamins, emerin and SUN proteins on softer matrices and glass coverslips for 90 mins. Zoom of single nucleus (inset) showing nucleoplasmic staining of these proteins on softer matrices. Arrowheads in panels Lamin A, Lamin B1 show altered nuclear morphologies, arrowheads in panel Emerin show extranuclear accumulation of emerin. (**C**–**H**) Normalized average fluorescence intensity from line-scans across nuclei performed for Lamin A (C), B1 (D), B2 (E), emerin (F), SUN1 (G) and SUN2 (H) in DLD-1 cells on softer matrices and glass after 90 min. Average intensities for each protein were normalized to their fluorescence intensities at the nuclear periphery in cells on glass (indicated by red dot) (pooled data from *N* = 2 independent biological replicates, *n*: number of nuclei, error bar: SEM). **P < 0.0001 (Student's *t*-test). (**J**) Immunostaining for Lamin A, B2 and emerin in cells switched from 2 kPa matrix to glass (representative 2 kPa images from B). (**K**) Normalized average fluorescence intensity from line-scans across nuclei performed for Lamin A, B2 and emerin in DLD-1 cells switched from 2 kPa matrix to glass. Average intensities for each protein were normalized to their fluorescence intensities at the nuclear periphery (indicated by red dot) (pooled data from *N* = 2 independent biological replicates, *n*: number of nuclei, error bar: SEM). **P < 0.05 (Student's *t*-test). Scale bar ∼10 μm.

We assessed the sub-nuclear localization of lamin/LINC proteins in single cells by confocal imaging (Figure 5A–B). Lamin/LINC proteins are typically localized at the nuclear envelope across cell types (94,95). Interestingly, cells exposed to softer matrices for ∼90 min, showed a distinctive mislocalization and enrichment of Lamin A, B1, B2, emerin, SUN1 and SUN2 into the nuclear interior in addition to their localization predominantly at the nuclear envelope in cells on glass (compare alternate panels in Figure 5B, B–H). Furthermore, cells on softer matrices showed distorted nuclear shapes—characteristic of nuclei with lowered lamin levels (arrowheads in Lamin A and B1 panels, Figure 5B) (17,41,93,96). Interestingly, emerin showed an extranuclear accumulation in ∼90% of cells, consistent with the mislocalization of emerin in cells with reduced lamin A/C levels (Arrows in emerin panel, Figure 5B and F) (97). Remarkably, Lamin A, Lamin B2 and emerin relocalized to the nuclear periphery in cells transferred from the softer matrices (2 kPa) to glass, along with a decrease in the extranuclear accumulation of emerin (Figure 5I–K, Supplementary Figure S6I). Taken together, the localization of nuclear envelope factors is remarkably sensitive to the stiffness of the extracellular matrix.

### Lamin B2 overexpression retains gene poor CT18 proximal to the nuclear periphery

**Figure 6.**
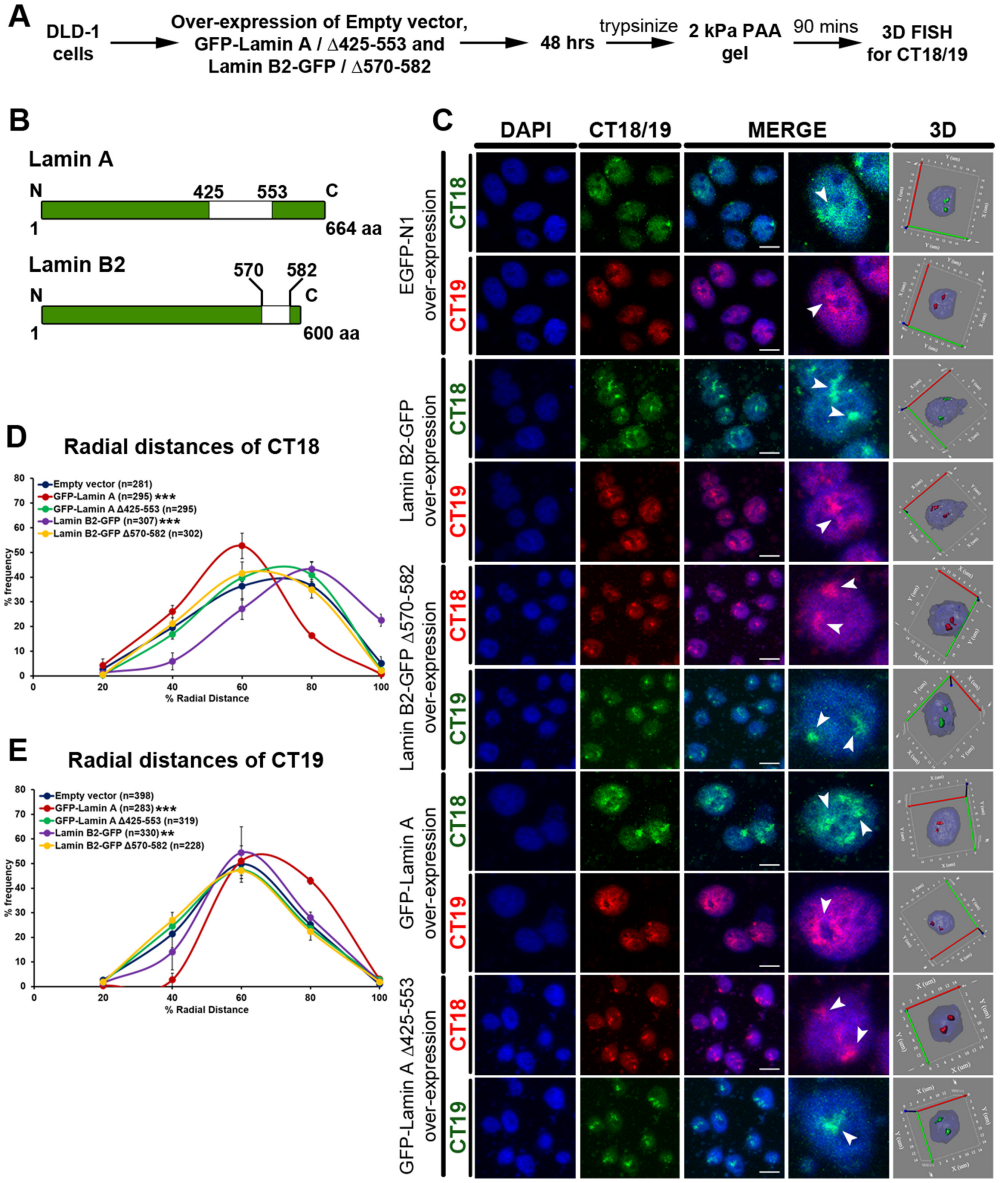
Lamin B2 overexpression retains gene poor CT18 proximal to the nuclear periphery. (**A**) Experimental scheme. (**B**) Graphical representation of mutants generated for Lamin A and B2. (**C**) Representative mid-optical sections from 3D-FISH hybridizations for CT18 and 19 in DLD-1 cells on 2 kPa matrix upon overexpression of Empty vector (EGFP-N1), Lamin A (GFP-Lamin A and GFP-Lamin A Δ425-553) and Lamin B2 (Lamin B2-GFP and Lamin B2-GFP Δ570–582). Arrowheads show specific hybridization for CT18 and CT19 resolved in 3D: reconstruction of single representative nucleus (*N* = 3 independent biological replicates). (**D**) Radial distance distribution profiles for CT18 on 2 kPa matrix upon over-expression of empty vector (*M* = 56.79%), GFP-Lamin A (*M* = 46.41%), GFP-Lamin A Δ425–553 (*M* = 57.08%), Lamin B2-GFP (*M* = 67.41%) and Lamin B2-GFP Δ570–582 (*M* = 54.07%). (**E**) Radial distance distribution profiles for CT19 on 2 kPa matrix upon over-expression of empty vector (*M* = 50.98%), GFP-Lamin A (*M* = 58.46%), GFP-Lamin A Δ425–553 (*M* = 48.73%), Lamin B2-GFP (*M* = 53.67%) and Lamin B2-GFP Δ570–582 (*M* = 48.93%) (D–E: pooled data from *N* = 3 independent biological replicates, *n*: number of CTs, X-axis: 0%—nuclear center and 100%—nuclear periphery, error bar: SEM, Mann–Whitney test). ***P < 0.0001, **P < 0.01 (compared with empty vector control). Scale bar ∼10 μm.

As Lamins mislocalized into the nuclear interior in cells on softer matrices (Figure 5B–E), we asked if lamin overexpression modulates chromosome positioning in the interphase nucleus (Supplementary Figure S7A-D). We performed 3D-FISH analyses to determine chromosome 18 and 19 territory positions in cells overexpressing either Lamin B2 or Lamin B2 Δaa570-582—as this region is predicted to be involved in chromatin association (Figure 6A–B) (98). Remarkably, cells on softer matrices (2 kPa) overexpressing the full length Lamin B2, retained CT18 closer toward the nuclear periphery (R.D ∼67.41%, empty vector: R.D ∼56.79%) (Figure 6C–D, Supplementary Figure S7E, Table 1, Supplementary Table S4). Of note, CT18 mislocalization toward the nuclear interior was unaffected in cells overexpressing Lamin B2Δ570-582 and exposed to softer matrices (2 kPa) (R.D ∼54.07%) (Figure 6C–D, Supplementary Figure S7E, Table 1, Supplementary Table S4). In summary, optimum levels of Lamin B2, its enrichment at the nuclear periphery and ability to interact with chromatin are potentially required for positioning CT18 closer to the nuclear periphery in cells on softer matrices. This further underscores the role of Lamins and their interaction with Lamina Associated Domains (LADs) in positioning CT18 toward the periphery of the interphase nucleus (33,99). In contrast, the internal nuclear localization of gene rich CT19, was unaffected in cells on the softer matrices (2 kPa) overexpressing either Lamin B2 (R.D ∼53.67%) or Lamin B2Δ570-582 (R.D ∼48.93%, Empty vector: R.D ∼50.98%) (Figure 6C and E, Supplementary Figure S7F, Table 1, Supplementary Table S4). Lamin A overexpressing cells on softer matrices mislocalized CT18 further into the nuclear interior (R.D ∼46.41%, Empty vector: R.D ∼56.79%) and CT19 away from the nuclear interior (R.D ∼58.46%, empty vector: R.D ∼50.98%) (Figure 6C–E, Supplementary Figure S7E–F, Table 1, Supplementary Table S4). However, Lamin AΔ425–553 overexpression (with reduced chromatin association, (98)) did not perturb the mislocalization of either CT18 (R.D ∼57.08%) or CT19 (R.D ∼48.73%) (Figure 6C–E, Supplementary Figure S7E–F, Table 1, Supplementary Table S4). While creating Lamin AΔ425–553, we retained the NLS sequence (aa 417–422) of Lamin A that interacts with histones, as its absence sequesters Lamin A/C into the endoplasmic reticulum (100).

Lamin overexpression also showed a marginal (but not significant) increase in the levels of Lamin A, B1, B2, phospho-emerin, SUN1 and SUN2 (Supplementary Figure S7G–H). Notably, overexpressed Lamins A and B2 were enriched at the nuclear envelope in cells on softer (2 kPa) matrices (Supplementary Figure S7C–D). Interestingly, Lamin A or B2 (full length and mutant) overexpression did not affect the relative localization of either CT18 or CT19 in cells on glass (Supplementary Figure S8A–G, Supplementary Table S4). In summary, chromosome territory positions are differentially responsive to Lamin A or B2 overexpression in cells on softer matrices.

### Inhibition of emerin phosphorylation selectively abrogates mislocalization of chromosome territories

**Figure 7.**
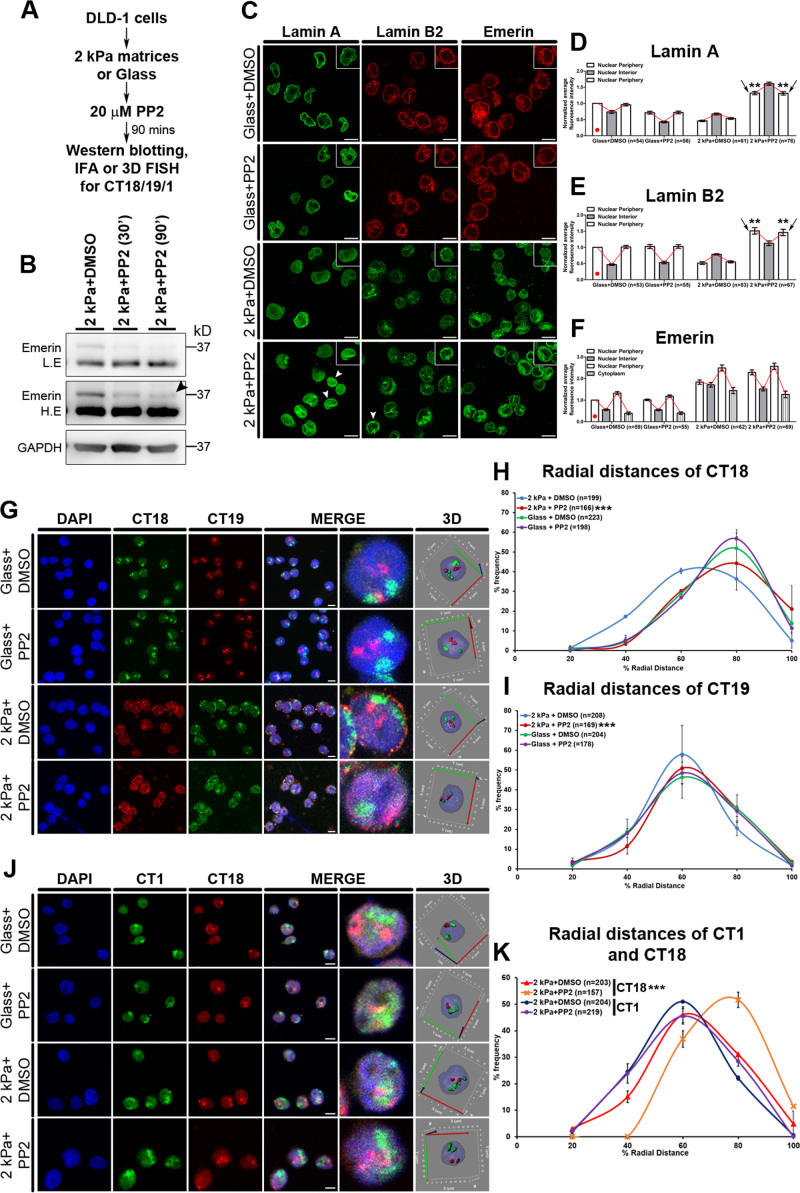
Inhibition of Emerin phosphorylation selectively abrogates chromosome territory movements. (**A**) Experimental scheme. (**B**) Western blot analysis to assess effect of PP2 treatment on emerin phosphorylation. Blot probed with anti-emerin antibody (upper panel—lower exposure (LE), lower panel—higher exposure (HE)) and GAPDH was used as loading control (*N* = 3). Arrowhead indicates significant reduction of phospho-emerin band upon 20 μM PP2 treatment for ∼90 min. (**C**) Representative mid-optical sections of DLD-1 cells immunostained for Lamin A, B2 and emerin on 2 kPa matrix and glass for 90 mins, with and without (DMSO control) 20 μM PP2. Inset: zoom of single nucleus. Arrowheads indicate increased Lamin A and B2 staining at the periphery on 2 kPa matrices with PP2 treatment. (**D**–**F**) Normalized average fluorescence intensity from line-scans across nuclei performed for Lamin A (D), Lamin B2 (E) and emerin (F) in DLD-1 cells on 2 kPa matrix and glass (with and without PP2) after 90 min. Average intensities for each protein were normalized to their fluorescence intensities at the nuclear periphery in cells on glass (indicated by red dot) (pooled data from *N* = 2 independent biological replicates, *n*: number of nuclei, error bar: SEM). **P < 0.0001 (Student's *t*-test). (**G**) Representative mid-optical sections from 3D-FISH hybridizations for CT18 and 19 on 2 kPa matrix and glass, with and without (DMSO control) 20 μM PP2 treatment. 3D: reconstruction of single representative nucleus. H) Radial distance distribution profiles for CT18 (*N* = 2) on 2 kPa matrix (+PP2: *M* = 66.83% and –PP2: *M* = 54.28%) and glass (+PP2: *M* = 67.93% and –PP2: *M* = 66.31%). (**I**) Radial distance distribution profiles for CT19 (*N* = 2) on 2 kPa (+PP2: *M* = 53.83% and –PP2: *M* = 48.93%) and glass (+PP2: 54.56% and –PP2: *M* = 53.15%) (H–I: Data pooled from *N* = 2 independent biological replicates, *n*: number of CTs, X-axis: 0%—nuclear center and 100%—nuclear periphery, error bar: SEM, Mann–Whitney test). ***P < 0.0001 (compared with 2 kPa+DMSO). (**J**) Representative mid-optical sections from 3D-FISH hybridizations for CT1 and 18 on 2 kPa matrix and glass, with and without (DMSO control) 20 μM PP2 treatment. 3D: reconstruction of single representative nucleus. (**K**) Radial distance distribution profiles of CT1 on 2 kPa (+PP2: *M* = 49.70% and –PP2: *M* = 50.94%) and CT18 on 2 kPa (+PP2: 65.73% and –PP2: *M* = 55.47%) (data pooled from *N* = 2 independent biological replicates, *n*: number of CTs, X-axis: 0%—nuclear center and 100%—nuclear periphery, error bar: SEM, Mann–Whitney test). ***P < 0.0001 (compared with 2 kPa + DMSO). Scale bar ∼10 μm.

Emerin is phosphorylated by Src kinase at Tyr74 and Tyr95 respectively in isolated nuclei deformed by magnetic tweezers (21). We examined the impact of inhibiting emerin phosphorylation by treating cells on softer matrices with PP2 (an inhibitor of the Src tyrosine kinase family, (101)) (Figure 7A–B). Interestingly, inhibition of Src kinase activity (and therefore emerin phosphorylation) increased and retained Lamin A and Lamin B2 at the nuclear envelope in cells on softer matrices (Figure 7C–F). Furthermore, neither CT18 nor CT19 mislocalized into the nuclear interior in cells on softer matrices (2 kPa), upon inhibition of emerin phosphorylation (CT18: 2 kPa + PP2 RD ∼66.83%; CT19: 2 kPa + PP2 RD ∼53.83%) (Figure 7G–I, Supplementary Figure S10A–B, Table 1, Supplementary Table S4). Taken together, this suggests a unique role for emerin phosphorylation in modulating the spatial positions of chromosome 18 and 19 territories in cells on softer matrices.

Surprisingly, the inhibition of emerin phosphorylation did not affect the position of CT1 that was mislocalized toward the nuclear interior in cells on softer matrices (2 kPa + DMSO: RD ∼50.94%, 2 kPa + PP2: RD ∼49.70%) (Figure 7J–K, Supplementary Figure S10C, Table 2, Supplementary Table S5). This was in marked contrast to CT18 and CT19 positions, that were retained at their conserved nuclear locations upon inhibition of emerin phosphorylation (Figure 7H–I). We determined the positions of CT18 along with CT1 by co-labeling these chromosome territories upon inhibition of emerin phosphorylation in cells on softer matrices (2 kPa). As observed previously, CT18 was retained toward the nuclear periphery upon inhibition of emerin phosphorylation (CT18: 2 kPa + DMSO: RD ∼55.47%, 2 kPa + PP2: RD ∼65.73%), while position of CT1 remained unaffected (Figure 7J–K, Supplementary Figure S10D). Taken together, these results suggest that the positions of chromosome territories are selectively responsive to emerin phosphorylation.

**Table 2.** Radial distance measurements for CT1 under conditions of altered matrix stiffness

	Median %radial distance (%RD)	
Substrate/conditions	CT1	Δ
(I) CT positions on softer matrices after 90 min (reference for comparison: glass)		
2 kPa (90 min)	**50.57 (*P*< 0.0001)**	0
Glass (90 min)	66.81	+16.24
(II) CT positions upon PP2 treatment on softer matrices (2 kPa) (Reference for comparison: respective DMSO control)		
Glass + DMSO	61.00	+10.43
Glass + 20 μM PP2	59.75	+9.18
2 kPa + DMSO	50.94	+0.37
2 kPa + 20 μM PP2	49.7	−0.87
(III) CT positions upon Emerin Y99F overexpression on softer matrices (2 kPa) (Reference for comparison: shEmerin+EGFP-N1)		
Vector control + EGFP-N1 on 2 kPa	**45.61 (*P*< 0.0001)**	−4.96
shEmerin + EGFP-N1 on 2 kPa	38.49	−12.08
shEmerin + WT-EMD on 2 kPa	**45.86 (*P* = 0.0005)**	−4.71
shEmerin + EMD Y99F on 2 kPa	**43.80 (*P* = 0.0015)**	−6.77

Median radial distances of CT1. Δ: shift in CT position, calculated with 2 kPa as reference, ‘+’: movement towards the nuclear periphery, ‘−’: movement towards the nuclear center. Values in bold are significant (*P* value in brackets).

### Emerin is phosphorylated at the Tyr99 residue in cells subjected to reduced matrix stiffness

**Figure 8.**
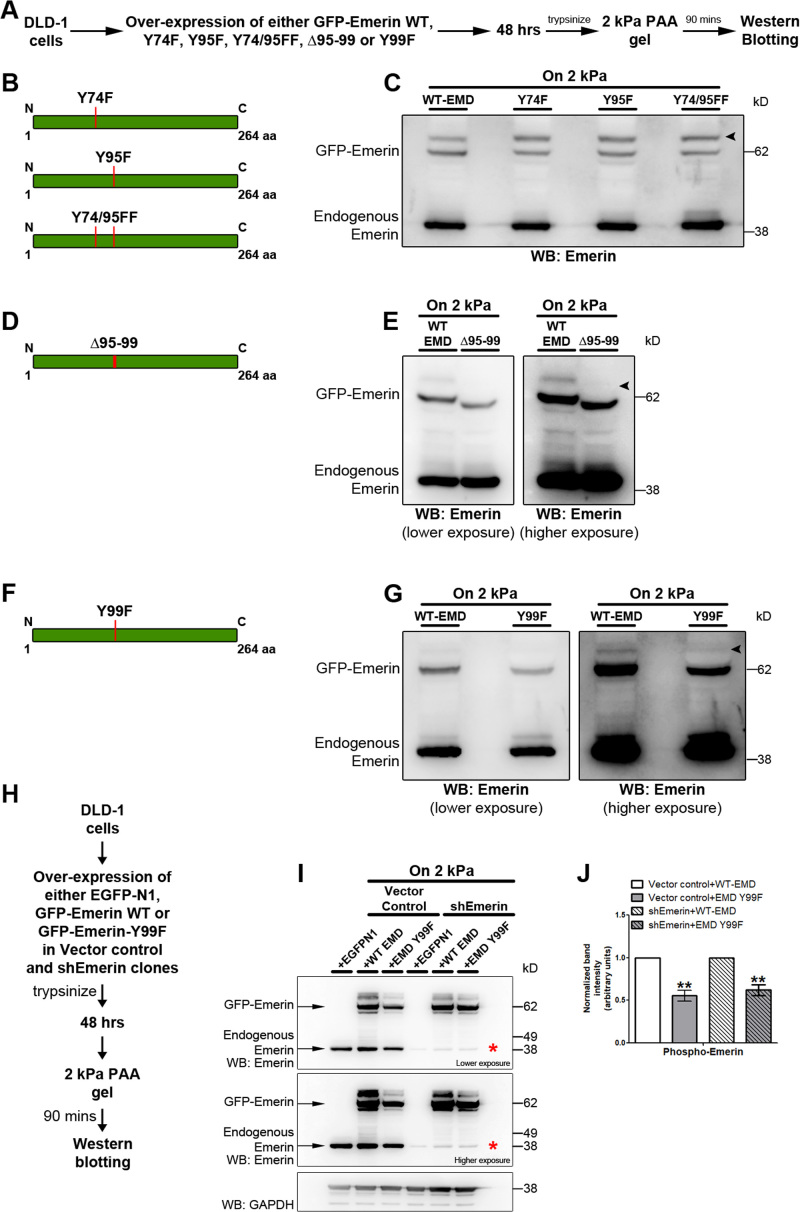
Emerin is phosphorylated at Tyr99 residue in cells subjected to reduced matrix stiffness. (**A**) Experimental scheme. (**B**) Graphical representation of site directed mutations in emerin (Y74F, Y95F, Y74/95FF). (**C**) Representative immunoblot to assess phosphorylation status of Wild Type (WT), Y74F, Y95F and Y74/95FF emerin upon over-expression for 48 hrs, followed by 90 min on 2 kPa matrix (*N* = 2). Arrowhead indicates phosphorylation of emerin Y74F, Y95F and Y74/95FF. (**D**) Graphical representation for emerin (Δ95–99). (**E**) Representative immunoblot to assess phosphorylation of WT and emerin (Δ95–99) upon over-expression for 48 h, followed by 90 min on 2 kPa matrix (*N* = 2). Arrowhead indicates reduced phosphorylation of overexpressed emerin (Δ95–99). (**F**) Graphical representation of site directed mutation (Y99F) in emerin. G) Representative immunoblot to assess phosphorylation of WT and Y99F emerin upon over-expression for 48 h, followed by 90 min on 2 kPa matrix (*N* = 2). Arrowhead indicates reduced phosphorylation of overexpressed emerin Y99F (for C, E and G: EMD: emerin, *N*: independent biological replicates). (**H**) Experimental scheme for overexpression of WT and Y99F Emerin in shEmerin background. (**I**) Representative immunoblot (*N* = 3 independent biological replicates) showing overexpression of GFP-Emerin WT and GFP-Emerin Y99F in vector control and shEmerin clones. Asterisk: Distinct downregulation of endogenous emerin in shEmerin clone. GAPDH was used as loading control. (**J**) Densitometric quantification of GFP-Emerin WT and Y99F phosphorylation in vector control and shEmerin clones. Overexpressed phosphorylated and non-phosphorylated emerin expression was normalized to GAPDH. The phosphorylated emerin levels were then re-normalized to total overexpressed emerin levels (pooled data from *N* = 3 independent biological replicates, error bars: SEM). **P< 0.01 (Student's *t*-test).

We sought to identify the tyrosine residue, phosphorylated in emerin in response to lowered matrix stiffness. Src kinase phosphorylates emerin at tyrosine residues (Y) 59, 74 and 95 (20). Of these, Y74 and Y95 are phosphorylated by Src in a force-dependent manner (21). We examined if the phospho-deficient mutants of emerin i.e, Y74F, Y95F and Y74/95FF were phosphorylated in cells on softer matrices (2 kPa) (Figure 8A–C). Interestingly, even these phospho-deficient emerin mutants were phosphorylated, comparable to overexpressed wild type emerin (Arrowhead, Figure 8C), suggesting that another tyrosine residue was phosphorylated in emerin. We next exposed cells over-expressing emerin (Δ95–99)—a mutant with reduced affinity to Lamin A/C (102), to the softer matrices (2 kPa) (Figure 8D–E). Western blotting showed a distinct reduction of emerin (Δ95–99) phosphorylation (Arrowhead, Figure 8E). Since emerin has another tyrosine residue at Tyr99, we tested the phosphorylation status of Y99 (Figure 8F–G). Interestingly, overexpressed emerin Y99F showed a distinctive decrease in phosphorylation levels in cells exposed to softer matrices (2 kPa) (Arrowhead, Figure 8G). It is noteworthy that the overexpressed phospho-deficient mutants of emerin (Y74F, Y95F, Y74/95FF and Y99F) were localized at the inner nuclear membrane and a sub-fraction outside the nucleus, comparable to overexpressed wild type (WT) emerin in cells on both soft (2 kPa) matrices and glass (Supplementary Figure S9A–C). Taken together, emerin phosphorylation at Tyr99 residue is activated in cells and functions as a key mechanosensitive signal in response to lowered matrix stiffness.

### Overexpression of emerin Y99F, selectively abrogates mislocalization of chromosome territories

**Figure 9.**
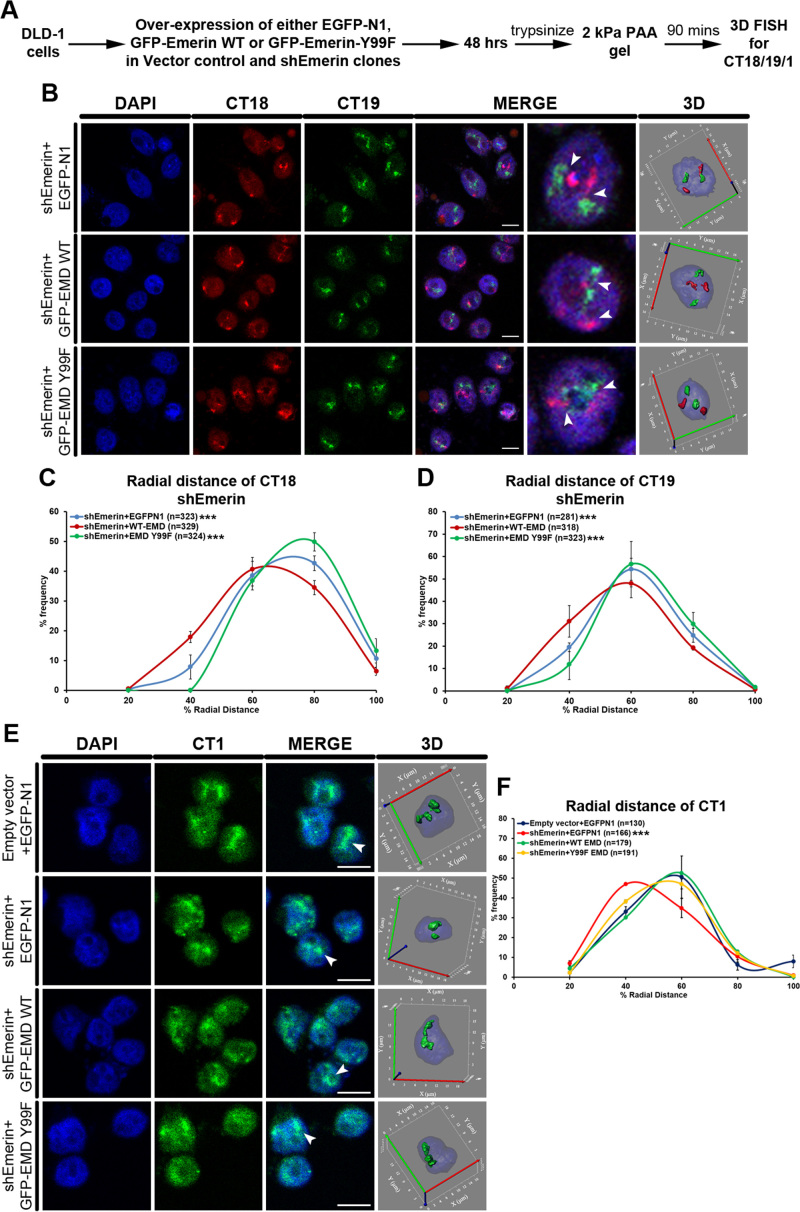
Overexpression of Emerin Y99F selectively abrogates mislocalization of chromosome territories. (**A**) Experimental scheme. (**B**) Representative mid-optical sections from 3D-FISH hybridizations for CT18 and 19 in shEmerin clones on 2 kPa matrix after overexpression of Empty vector (EGFP-N1), WT Emerin (GFP-EMD WT) and Emerin Y99F (GFP-EMD Y99F). Arrowheads show specific hybridization for CT18 and CT19, resolved in 3D: reconstruction of single representative nucleus. (**C**) Radial distance distribution profiles for CT18 in shEmerin clone (on 2 kPa) after over-expression of Empty vector (*N* = 3, *M* = 61.80%), GFP-EMD WT (*N* = 3, *M* = 55.72%) and GFP-EMD Y99F (*N* = 3, *M* = 65.25%). (**D**) Radial distance distribution profiles for CT19 in shEmerin clone (on 2 kPa) after over-expression of Empty vector (*N* = 3, *M* = 51.13%), GFP-EMD WT (*N* = 3, *M* = 47.50%) and GFP-EMD Y99F (*N* = 3, *M* = 53.24%) (B–D, EMD: emerin, pooled data from *N* = 3 independent biological replicates, *n*: number of CTs, X-axis: 0%—nuclear center and 100%—nuclear periphery, error bar: SEM, Mann–Whitney test). ***P< 0.0001 (compared with Vector control+EGFP-N1, Supplementary Figure S9A–D). (**E**) Representative mid-optical sections from 3D-FISH hybridizations for CT1 in vector control clone on 2 kPa matrix after overexpression of empty vector (EGFP-N1), and shEmerin clones on 2 kPa matrix after overexpression of empty vector (EGFP-N1), WT Emerin (GFP-EMD WT) and Emerin Y99F (GFP-EMD Y99F). Arrowheads show specific hybridization for CT1, resolved in 3D: reconstruction of single representative nucleus. (**F**) Radial distance distribution profiles for CT1 in vector control clone (on 2 kPa) after overexpression of empty vector (*N* = 2, *M* = 45.61%), and shEmerin clone (on 2 kPa) after over-expression of empty vector (*N* = 2, *M* = 38.49%), GFP-EMD WT (*N* = 2, *M* = 45.86%) and GFP-EMD Y99F (*N* = 2, *M* = 43.80%) (E–F, EMD: emerin, pooled data from *N* = 2 independent biological replicates, *n*: number of CTs, X-axis: 0%—nuclear center and 100%—nuclear periphery, error bar: SEM, Mann–Whitney test). ***P< 0.0001 (compared with vector control+EGFP-N1). Scale bar ∼10 μm.

Since emerin Y99F showed a distinctive reduction in its phosphorylation in cells on softer matrices, we asked if emerin Y99 that functions as a mechanosensor of reduced matrix stiffness, modulates chromosome territory positions? We depleted cells of endogenous emerin using emerin shRNA to generate sub-clones of cells with lowered emerin levels (Figure 8H–J, Supplementary Figure S10E). Interestingly, cells depleted of endogenous emerin (shEmerin) showed a relatively conserved positioning of CT18 toward the periphery (RD ∼61.80%) and CT19 near the nuclear interior (RD ∼51.13%) on softer matrices (Figure 9A–D, Supplementary Figure S11A–F, Table 1, Supplementary Table S4). In addition, upon depletion of emerin, Lamin A and B2 levels were elevated in cells on softer matrices (Supplementary Figure S10F–H). Taken together, this suggests the fundamental requirement of emerin and consequently emerin phosphorylation in the mislocalization of chromosome 18 and 19 territories in cells exposed to softer matrices. Consistent with these results, X-EDMD patient fibroblasts expressing mutant emerin, mislocalize chromosome 13 and 18 territories toward the nuclear interior (43), while in contrast lymphocytes with a nonsense mutation in emerin, and therefore emerin loss, nevertheless retained conserved radial positioning of chromosome territories (103).

We overexpressed either wild type emerin or emerin Y99F (both insensitive to emerin shRNA) in sub-clones depleted of endogenous emerin (shEmerin) and vector control clones for 48 h, followed by exposing these cells to softer matrices (2 kPa) for ∼90 min (Figures 8H–J and 9A–B, Supplementary Figure S11A–B). Emerin depleted cells overexpressing emerin Y99F (on 2 kPa), showed a significantly greater retention of Lamin A and B2 at the nuclear envelope (Supplementary Figure S10F–H). Remarkably, CT18 and CT19 were also retained at their conserved nuclear locations in cells overexpressing emerin Y99F (but depleted of endogenous emerin, CT18: RD ∼65.25%; CT19: RD ∼53.24%) (Figure 9A–D, Supplementary Figure S11A–F, Table 1, Supplementary Table S4). In contrast, the overexpression of WT emerin (in emerin depleted cells) mislocalized CT18 and CT19 on softer matrices (CT18: RD ∼55.72%; CT19: RD ∼47.50%) (Figure 9A–D, Supplementary Figure S11A-F, Table 1, Supplementary Table S4). Furthermore, Lamin A and B2 were predominantly enriched in the nucleoplasm rather than the nuclear envelope, upon overexpression of WT emerin (Supplementary Figure S10F–H). This further underscores the requirement of emerin phosphorylation in modulating chromosome 18 and 19 territory positions in cells on softer matrices.

Since CT1 was mislocalized toward the nuclear interior despite the inhibition of emerin phosphorylation (Figure 7J–K), we determined the effect of overexpressing emerin Y99F on CT1 localization (Figure 9E–F). Interestingly, cells depleted of endogenous emerin (shEmerin) mislocalized CT1 even further into the nuclear interior (Vector control clone+EGFP-N1: RD ∼45.61%, shEmerin+EGFP-N1: RD ∼38.49%) (Figure 9E–F, Supplementary Figure S11G, Table 2, Supplementary Table S5). Remarkably, overexpression of either WT emerin or emerin Y99F (in shEmerin background) rescued CT1 positions only comparable to that of control cells (Vector control clone+EGFP-N1: RD ∼45.61%, shEmerin+EGFP-N1: RD ∼38.49%, +WT-EMD: RD ∼45.86%, +EMD Y99F: RD ∼43.80%) (Figure 9E–F, Supplementary Figure S11G, Table 2, Supplementary Table S5). In summary, CT1 mislocalization toward the nuclear interior is independent of emerin phosphorylation in cells on softer matrices, while its localization is emerin dependent. Taken together, chromosome territory positions are selectively responsive to the phosphorylation status of emerin.

## DISCUSSION

Chromosome positions are conserved in the interphase nucleus in a gene density dependent manner (23–25,54). However, chromosome territory positions are altered during (i) adipocyte and myogenic differentiation (26,29) (ii) spermatogenesis (31) (iii) quiescence or senescence (27), and (iv) DNA damage response, mediated by the nuclear motor—nuclear myosin I (28,104). Chromosome territories are repositioned within ∼15 min in serum starved cells, or in a few hours upon DNA damage induction and after days during cell differentiation. This suggests that a dynamic but cell type and context specific response, when relayed to the nucleus, repositions chromosome territories.

**Figure 10.**
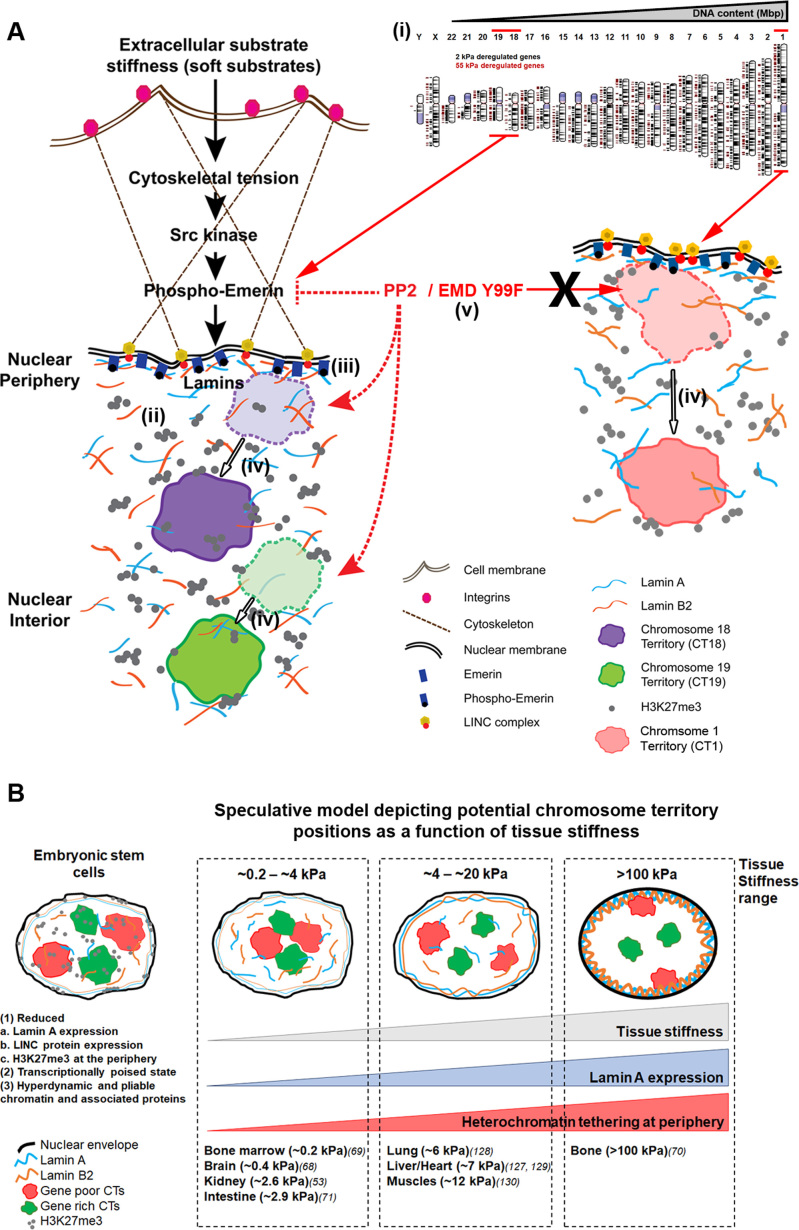
Emerin phosphorylation signals the mislocalization of chromosome territories in cells exposed to softer matrices. (**A**) Cells in contact with softer matrices show (i) altered transcriptional profiles (ii) nucleoplasmic accumulation of Lamin/LINC complex proteins and inactive histone marks (H3K27me3) (iii) activation of emerin phosphorylation at Tyr99 (iv) Chromosome territories CT1, CT18 and 19 are collectively mislocalized toward the nuclear interior upon lowered matrix stiffness (v) Inhibition of emerin phosphorylation by Src kinase inhibitor (PP2) or overexpression of phospho-deficient emerin Y99F in cells on softer matrices retains Lamins at the nuclear envelope, CT18 and 19 at their conserved nuclear locations, but not CT1. Emerin phosphorylation is a key upstream mechanosensor of lowered matrix stiffness that selectively modulates chromosome territory positions in cells on softer matrices. (**B**) Speculative model depicting potential chromosome territory positions as a function of tissue stiffness. Embryonic stem cells (ES cells) have a ‘floppier’ chromatin architecture in the interphase nucleus. We speculate that cells in softer tissues have a relaxed organization of chromosome territories, that are reorganized and adopt more rigid configurations as a function of increased lamin levels and stiffness of the extracellular matrix.

Here, we show that chromosome territories are repositioned within a relatively short duration of ∼90 min, in cells exposed to softer extracellular matrices. Interestingly, the mislocalization of chromosome territories was dependent on emerin, but was differentially sensitive to emerin phosphorylation at Tyr99 (Figure 9). This is consistent with an established role of emerin as a mechanosensor, as its Tyr74 and 95 residues are phosphorylated in response to increased nuclear strain (21). Here, we identified an additional residue on emerin (Tyr99), phosphorylated in response to reduced cellular strain, which also alters lamin localization accompanied by the selective mislocalization of chromosomes 18 and 19 but not chromosome 1 territories in the interphase nucleus (Figure 10A).

### Transcriptional deregulation largely correlates with chromosome repositioning in response to reduced matrix stiffness

Substrate stiffness is a well-known modulator of gene expression (52,73–76). For instance, PtK2 epithelial cells show increased levels of Heterochromatin Protein 1β (HP1β), heterochromatinization and transcriptional repression on softer extracellular matrices (<50 kPa) (50,51). Interestingly, RNA-Seq analyses of cells on softer matrices revealed a comparable extent of up and down regulated genes on the matrices (Figure 1B–C).

Remarkably, Chr. 1 showed maximum transcriptional deregulation (Figure 1E, Supplementary Figure S2A, C and E). Interestingly, although Chr. 18 showed a total deregulation of ∼6%, CT18 shifted significantly toward the nuclear interior by a radial distance of ∼10% (1.013 ± 0.13 μm). While Chr. 1 and 19 with ∼16.64% and ∼11.83% deregulation mislocalized toward the nuclear interior by a radial distance of ∼16% (1.74 ± 0.1 μm) and ∼5% (0.42 ± 0.13 μm) respectively in cells on softer matrices (2 kPa) (Figures 1E and 2C–E, Supplementary Figure S2C and E, Table 1). In summary, this analysis suggests that the extent of chromosome territory mislocalization does not necessarily correlate with the extent of its transcriptional deregulation in cells subjected to reduced mechanical stress.

In particular, the mislocalization of CT1 on softer matrices was independent of emerin phosphorylation at Tyr99 (in contrast to CT18 and 19) (Figure 9C–D and F). However, CT1 mislocalized further into the nuclear interior in the absence of endogenous emerin (shEmerin cells), while CT18 and 19 were retained at their conserved nuclear locations (Figure 9C–D and F). This suggests that emerin modulates chromosome territory positions in cells subjected to reduced matrix stiffness. Consistent with these results, human Chr. 1 is transcriptionally deregulated and shows altered nuclear positions in BJ-1 fibroblasts subjected to longitudinal micropatterns (49). Furthermore, CT1 (harboring epidermal differentiation cluster) showed a significant internal nuclear localization in epidermal progenitor cells subjected to biaxial cyclic mechanical strain (44). These studies highlight CT1 as a unique responder to altered mechanical equilibrium of cells, consistent with its striking transcriptional imbalance as well as its mislocalization in cells exposed to soft matrices (Figures 1E and 2C). We speculate that the following properties of human chromosome 1 namely (i) DNA content (ii) a unique 3D topology in the interphase nucleus (iii) its extensive association with Lamins and their interactors like Lap2α, BAF among others and (iv) transcriptional status, collectively contribute to the nuclear dynamics of chromosome 1 territory. Emerin phosphorylation at Tyr99 is required for the selective mislocalization of CT18 and 19 toward the nuclear interior, but not CT1 (Figure 9C–D and F), which further underscores the differential sensitivity of chromosome territories to specific external mechanical stimuli.

### Lamins as effectors of chromosome territory positions

It is noteworthy that the spatial position of chromosome 18 territory, closer to the nuclear periphery, was considerably more sensitive to altered matrix stiffness as compared to gene rich CT19 toward the nuclear interior (Figure 3C–D). Additionally, repositioning of gene poor CT18 (peripheral) toward the nuclear interior is accompanied by the mislocalization of Lamins/LINC factors and the inactive histone mark H3K27me3 to the nuclear interior, otherwise associated with heterochromatin at the nuclear periphery (Figures 1K–L, 5B–H). This suggests that lamin mediated heterochromatin association maintains CT18 and potentially positions of other gene poor chromosome territories toward the nuclear periphery (Figure 6D). We speculate that the untethering of gene poor CTs such as CT1 and CT18 from the nuclear periphery is an early event in response to altered force perception preferentially at the nuclear envelope, by virtue of their relative proximity to the nuclear envelope and enrichment of LADs (23,33). A distinct compartment of repressed chromatin – perinucleolar heterochromatin associated with the nucleolus, is localized relatively closer to the nuclear interior and certain gene loci and LADs stochastically associate with the nucleolus post mitosis (105–107). Since Lamin sub-pools exist at the nucleolar border, we surmise that mislocalized chromosome territories may employ the nucleolus as a landmark during their repositioning relatively into the nuclear interior in cells on softer matrices (108–110). Of note, mislocalization of chromosomes 1, 18 and 19 territories into the nuclear interior also decreases their relative spatial separation, further suggesting a relaxation of conserved CT positions as a function of altered mechanical forces perceived by cells (Table 1).

Notwithstanding the comparable extent of emerin phosphorylation on both the softer matrices (Figure 4D), CT18 positions were significantly different between the two softer matrices (Figure 3C–D and Supplementary Figure S6C–F). It is likely that the differences in the levels of nuclear envelope proteins owing to altered substrate stiffness, in turn function as effectors of mechanosensitive responses in the nucleus. Lamin A/C, B1 and B2 are interdependent for their assembly into higher order structures at the nuclear lamina (111,112). In addition, localization and organization of LINC complex proteins (SUN1, SUN2, Nesprin-1α, Nesprin-2) is also dependent on Lamin A, suggesting a cross talk between lamins and nuclear envelope factors that determine the functional organization of the nuclear envelope (7,9,113–115). We surmise that a decrease in the levels of lamin/LINC proteins destabilizes the nuclear envelope, further contributing to their mislocalization into the nuclear interior (Figures 4A–B and 5B–H). Furthermore, chromosome positioning on softer matrices is dependent on the levels and DNA binding ability of Lamin A and Lamin B2 (Figure 6). Although, Lamin A or B2 overexpression did not affect emerin phosphorylation (Supplementary Figure S7G–H), chromosome 18 nevertheless remained proximal to the nuclear periphery upon overexpression of Lamin B2 in cells on softer matrices (Figure 6C–D). The chromatin binding domains of Lamin A and B2 are likely to also modulate protein-protein interactions with Lap2α, Histones, Emerin, Actin among others (116). This implies that lamin stoichiometry and interactions impinge on chromosome territory positions (112). B-type Lamins interact with LADs in heterochromatin via LBR and HP1α, consistent with a key regulatory role of Lamin B1/B-type Lamins in the positioning of gene poor chromosome territories (35,117,118). Lamin A/C, on the other hand, interacts with LADs at the nuclear periphery, and in the nuclear interior via its interaction with LAP2α (119–121). Thus, overexpression of Lamin A alters the spatial coordinates of chromosome territories, while Lamin B2 overexpression provides positional cues and positions the gene poor CT18 closer to the nuclear periphery (Figure 6D–E).

### Emerin as an upstream signal that modulates chromosome territory positions

Lamin A is phosphorylated at Ser22 and shows increased nucleoplasmic localization in response to reduced extracellular matrix stiffness (16,17). Our data suggests that in addition to Lamin A, levels of Lamin B1, B2, SUN1 and SUN2 are reduced and mislocalized into the nuclear interior in cells on softer matrices, underscoring that the nuclear envelope is highly perceptive and sensitive to external force transitions (Figures 4A–B, 5B–E, G–H). We speculate that emerin phosphorylation functions as an upstream regulator of lamin localization (Supplementary Figure S10F-H). Furthermore, the Tyr99 residue of emerin phosphorylated on softer matrices reported here, maps to the interaction domain between emerin and Lamin A/C (122–124). Substrate stiffness dependent phosphorylation of emerin by Src kinase may perturb emerin-Lamin A/C interaction, resulting in the mislocalization of Lamin A into the nucleoplasm and a sub-population of emerin outside the nucleus (Figure 5B–C, F) (125). However, the spatiotemporal regulation of emerin phosphorylation, lamin/LINC localization and their role in modulating chromosome organization and transcription remains largely unclear. Furthermore, the effect of Lamin/LINC conformations, polymerization, membrane associations and posttranslational modifications in transducing mechanical signals into the nucleus and chromatin that elicit context specific gene expression signatures, will prove to be pivotal to unravel the mechanisms that regulate mechanosignalling and transcription (16,17,126).

### Implications of a softer milieu on nuclear structure-function relationships

Lamin A expression levels positively correlate with an increase in tissue stiffness from as low as ∼0.2 kPa (brain) to >40 kPa (bone) (17,18,127–130,53,55,67–72). We propose a speculative model wherein gene poor chromosome territories and heterochromatin organization are relatively more relaxed in cells within softer tissues, consistent with lowered lamin levels (Figure 10B). In marked contrast, euchromatin or gene rich chromosome territories with (i) reduced LAD association (33) (ii) proximity to the nuclear interior (25) (iii) relatively greater number of housekeeping genes (131), are less responsive to changes in extracellular matrix stiffness. DLD-1 cells on softer matrices mimic nuclear organization of human and murine embryonic stem cells in terms of comparatively lowered lamin/LINC levels and nucleoplasm enrichment of H3K27me3 (132–137). Interestingly, the ‘softer’ nucleus of stem cells correlates with (i) increased ‘floppiness’ and plasticity of chromatin (138–142) (ii) reduced lamin levels (134,135) (iii) enhanced localization of H3K27me3 at the nuclear interior, reduction in H3K4me3 and a transcriptionally poised state (143,144). The decrease and redistribution of the nuclear envelope factors, i.e. lamin/LINC proteins into the nucleoplasm is likely to further contribute to transcriptional attenuation (145,146). This reiterates the fundamental role of nuclear envelope proteins in the regulation and maintenance of chromatin organization in differentiated cells and is consistent with elevated levels of A- and B-type Lamins during organogenesis that further establish non-random chromatin organization in differentiated cells (147). It remains to be determined if differential levels of phospho-emerin/emerin are modulators of chromosome positioning in a tissue stiffness dependent manner.

Taken together, these studies reveal that a mechanoresponsive role of nuclear envelope proteins impinge on the spatio-functional dynamics of chromosome territories in the interphase nucleus. The functional significance of chromosome positioning in terms of potentially altered chromatin contacts and its impact on the transcriptome remains to be examined in a cell-type and tissue specific context. It is therefore beyond doubt that elucidating the mechanisms that regulate mechanotransduction into the nucleus, will have far reaching consequences in understanding mechanobiology of tissue stiffness and its impact on diseases such as cardiomyopathies, muscular dystrophies and cancers.

## DATA AVAILABILITY

All datasets have been deposited in GEO. The GEO Accession number of the data set is: GSE108907.

## Supplementary Material

Supplementary Data
