# Supplementary material for: Emerin modulates spatial organization of chromosome territories in cells on softer matrices
Source: Nucleic Acids Res. 2018 Apr 19;46(11):5561–86. doi: 10.1093/nar/gky288 (PMC6009696; doi:10.1093/nar/gky288)
Supplement: Supplementary Data [file gky288_supplemental_files.zip › Supplementary Information_4th April 2018.docx]

**Supplementary Information**

**Supplementary Figure Legends**

**Figure S1.** **Cell and nuclear surface area changes on soft matrices plateau by ~90 minutes**

A) Validation of DLD-1 cell line by karyotyping metaphase chromosomes. DLD-1 cells are near diploid with modal chromosome number of 44-46. *(Inset)* Representative metaphase spread of DLD-1 cells (inverted DAPI, n=153 independent metaphases compiled from 3 independent biological replicates)

B) DLD-1 cells stained with phalloidin and DAPI. Cells were plated on softer matrices from ~15 min upto ~21 hrs

C-F) Dot scatter plots with median nuclear and cell areas respectively for DLD-1 cells on 2 kPa (C, D) and on 55 kPa matrices (E, F) across time. Area comparisons were performed between successive time points. *(Pooled data from N=3 independent biological replicates, n: number of cells).* *** p<0.001, ** p<0.01, * p<0.05 (Mann Whitney test). Cell and nuclear surface area increases as a function of time on softer matrices and plateau by ~90 minutes.

G-I) FACS analyses reiterating a predominant diploid (2n) status of DLD-1 cells on softer matrices 2 kPa (G), 55 kPa (H) and glass (I) at ~90 mins. Cell cycle profiles were unaltered on softer matrices at ~90 minutes. Scale bar ~10 µm.

**Figure S2. Transcriptional deregulation and lowering of H3K4me3 (active histone mark) is induced in cells on softer matrices**

A) Bar graph depicting %enrichment of deregulated genes (up and down together) in cells on 2

kPa matrices, on all the chromosomes. ≥ log 2-fold deregulated genes (up and down together –

1655 genes) were classified into bins of fold change (2-4 fold, 4-8 fold, 8-10 fold and >10 fold)

and mapped onto chromosomes. (Arrow) Chromosome 1 shows the maximum enrichment of

deregulated genes on 2 kPa (~33.5%). (Black box) Chromosome 18 is amongst the chromosomes

showing least enrichment of transcriptionally deregulated genes, while chromosome 19 is

amongst the chromosomes showing high enrichment.

B) Bar graph depicting %enrichment of deregulated genes (up and down together) in cells on 55

kPa matrices, on all the chromosomes. ≥ log 2-fold deregulated genes (up and down together –

1432 genes) were classified into bins of fold change (2-4 fold, 4-8 fold, 8-10 fold and >10 fold)

and mapped onto chromosomes. (Arrow) Chromosome 1 shows enrichment of deregulated genes on 55 kPa (~19.2%) to a much lesser extent than on 2 kPa (~33.5%). (Black box) Chromosome

18 is amongst the chromosomes showing least enrichment of transcriptionally deregulated genes,

while chromosome 19 is amongst the chromosomes showing high enrichment.

C) Bar graph depicting %­enrichment of up and downregulated genes (≥ log 2-fold up – 783 genes, down – 872 genes) in cells on 2 kPa matrices, on all the chromosomes.

D) Bar graph depicting %­enrichment of up and downregulated genes (≥ log 2-fold up – 649 genes, down – 783 genes) in cells on 55 kPa matrices, on all the chromosomes.

E) Bar graph depicting % up and downregulation in cells on 2 kPa matrices, on all the chromosomes. ≥ log 2-fold deregulated genes (up and down separately) on each chromosome were normalized to the total number of transcribing genes (FPKM>1) on that chromosome. *(Arrow)* Chromosome 1 shows the maximum deregulation on 2 kPa (~16.64%). *(Red box)* Chromosome 18 shows less transcriptional deregulation as compared to chromosome 19.

F) Bar graph depicting % up and downregulation in cells on 55 kPa matrices, on all the chromosomes. ≥ log 2-fold deregulated genes (up and down separately) on each chromosome were normalized to the total number of transcribing genes (FPKM>1) on that chromosome. *(Red box)* Chromosome 18 shows less transcriptional deregulation as compared to chromosome 19.

G) *(Left)* Representative western blots *(N=3)* for H3K4me3 and H3K27me3 expression levels in DLD-1 cells on soft matrices (2 kPa and 55 kPa) and tissue culture (TC) plastic after 90 mins. *(Right)* Representative western blots *(N=3)* for H3K4me3 and H3K27me3 expression levels in DLD-1 cells on softer matrices (2 kPa and 55 kPa) and upon switching cells from softer matrices to glass (90 mins). Pan Histone H3 was used as loading control.

H) Densitometric quantification of H3K4me3 and H3K27me3 levels on softer matrices (2 kPa and 55 kPa) and TC plastic after 90 mins. *(Pooled data from N=3 independent biological replicates).* * p<0.05 (Student’s t-test). H3K4me3 levels are significantly reduced in cells on 2 kPa matrices. No change is observed in levels of H3K27me3.

I) Densitometric quantification of H3K4me3 and H3K27 me3 levels on softer matrices (2 kPa and 55 kPa) and upon switching cells from softer matrices to glass (90 mins). *(Pooled data from N=3 independent biological replicates).* * p<0.05 (Student’s t-test). The reduced levels of H3K4me3 on softer matrices are recovered upon switching cells to glass. H3K27me3 levels are unchanged in all the conditions.

J) Normalized average total fluorescence intensity of H3K27me3 (images from Fig. 1K) in cells on softer matrices and glass for 90 minutes, and upon switch from 2 kPa to glass. Total average fluorescence intensity is comparable in all the above conditions *(Pooled data from N=2 independent biological replicates, Student’s t-test).*

**Figure S3. Classification of ≥ log 2-fold up and downregulated genes on 2 kPa matrices into Gene Ontology (GO) categories**

A) GO category classification of genes upregulated (≥ log 2-fold) in cells on 2 kPa matrices. Red boxes indicate the GO categories that are closely related to i) Cell cycle ii) Chromatin associated processes and iii) DNA damage and repair. Genes mapping to the GO categories of Cell cycle, RNA binding and DNA damage and repair are listed below.

B) GO category classification of genes downregulated (≥ log 2-fold) in cells on 2 kPa matrices. Red boxes indicate the GO categories that are closely related to i) Chromatin associated processes and ii) DNA damage and repair. Genes mapping to the GO categories of RNA binding and DNA damage and repair are listed below.

**Figure S4. Classification of ≥ log 2-fold up and downregulated genes on 55 kPa matrices into Gene Ontology (GO) categories**

A) GO category classification of genes upregulated (≥ log 2-fold) in cells on 55 kPa matrices. Red boxes indicate the GO categories that are closely related to i) GTPase signaling ii) Chromatin associated processes iii) Cell cycle and iv) Cytoskeleton and cell junctions. Genes mapping to the GO categories of Rho GTPase binding, DNA binding, Cell cycle and Actomyosin structure and organization are listed below.

B) GO category classification of genes downregulated (≥ log 2-fold) in cells on 55 kPa matrices. Red boxes indicate the GO categories that are closely related to i) Chromatin associated processes ii) DNA damage and repair and iii) Cell cycle. Genes mapping to the GO categories of DNA binding, DNA damage and repair and Cell cycle are listed below.

**Figure S5. Chromosome territories (18 and 19) are mislocalized towards the nuclear interior even upon prolonged exposure to softer matrices**

A) Dot scatter plot with median (M) % R.D for CT18 on softer matrices and glass across 3 independent biological replicates after 90 mins *(n: number of each CT, 0% - Nuclear center and 100% - Nuclear periphery).*

B) Dot scatter plot with median (M) % R.D for CT19 on softer matrices and glass across 3 independent biological replicates after 90 mins *(n: number of each CT, 0% - Nuclear center and 100% - Nuclear periphery).*

C) Dot scatter plot with median (M) % R.D for CT1 on soft (2 kPa) matrices and glass across 2 independent biological replicates after 90 mins *(n: number of each CT, 0% - Nuclear center and 100% - Nuclear periphery).*

D) Representative mid-optical section from 3D-FISH hybridization of CT18 and CT19 in DLD-1 cells exposed to 2 kPa matrix and glass for ~7 hours.

E) Dot scatter plot with median (M) % R.D values for CT18 on 2 kPa matrix (M=55.91%) and glass (M=66.73%) after ~7 hours *(N=2 independent biological replicates)*. *** p<0.0001 (Mann Whitney test). CT18 positions on 2 kPa matrices are significantly mislocalized near the nuclear interior at ~7 hrs.

F) Dot scatter plot with median (M) % R.D values for CT19 on 2 kPa matrix (M=45.30%) and glass (M=54.30%) after 7 hours *(N=2 independent biological replicates)*. *** p<0.0001 (Mann Whitney test). CT19 positions on 2 kPa matrices are significantly mislocalized near the nuclear interior at ~7 hrs.

G) Representative mid-optical section from 3D-FISH hybridization for CT18 and CT19 in DLD-1 cells exposed to 2 kPa matrix and glass for ~21 hours.

H) Dot scatter plot with median (M) % R.D values for CT18 on 2 kPa matrix (M=56.04%) and glass (M=66.93%) after 21 hours *(N=2 independent biological replicates)*. *** p<0.0001 (Mann Whitney test). CT18 positions on 2 kPa matrices are significantly mislocalized near the nuclear interior at ~21 hrs.

I) Dot scatter plot with median (M) % R.D values for CT19 on 2 kPa matrix (M=45.42%) and glass (M=53.81%) after 21 hours *(N=2 independent biological replicates)*. *** p<0.0001 (Mann Whitney test). CT19 positions on 2 kPa matrices are significantly mislocalized near the nuclear interior at ~21 hrs. Scale bar ~10 µm.

**Figure S6.**

A-B) Nuclear topologies in cells on softer matrices - Dot scatter plots with median (M) values for surface area and volume respectively of nucleus on softer matrices and glass after 90 mins, and upon the matrix switching assay *(n: number of nuclei, Pooled data from N=2 independent biological replicates).*

C) *(For C-F)* Experimental scheme for matrix switching experiment between softer matrices

D) Representative mid-optical sections from 3D-FISH hybridization for CT18 and CT19 in DLD-1 cells switched between the ­softer matrices.

E) Representative dot scatter plot with median (M) % R.D values for CT18 on 2 kPa (M=54.36%), 55 kPa (M=60.41%) and in cells switched between softer matrices (55 kPa to 2 kPa, M=51.52% and 2 kPa to 55 kPa, M=58.59%) *(N=2 independent biological replicates).* ** p<0.001 (Mann Whitney test).

F) Representative dot scatter plot with median (M) % R.D values for CT19 on 2 kPa (M=49.97%), 55 kPa (M=53.27%) and in cells switched between softer matrices (55 kPa to 2 kPa, M=50.25% and 2 kPa to 55 kPa, M=47.05%) *(N=2 independent biological replicates).* *** p<0.0001, * p<0.05 (Mann Whitney test). Scale bar ~10 µm.

G) Representative western blot *(N=2)* for emerin expression levels on softer matrices at 90 mins. Blots were probed with anti-emerin and anti Phospho-tyrosine antibodies. Arrowhead indicates tyrosine-phosphorylated form of emerin. GAPDH was used as loading control.

H) Representative western blots *(N=2)* showing emerin phosphorylation levels in DLD-1 cells on softer matrices after 7 and 21 hrs. GAPDH was used as loading control. Red dot indicates phosphorylated emerin*,* which is sustained in cells on softer matrices upto ~7 hrs but shows reduced levels at longer time points of ~ 21 hrs.

I) Immunostaining for Lamin A, B2 and Emerin in cells switched from 2 kPa matrix to glass (from Figure 5J). Scale bar ~10 µm.

**Figure S7. Lamin A and B2 overexpression on softer (2 kPa) matrices**

A) Bright-field and fluorescence images of DLD-1 cells (acquired at 20X objective, Invitrogen EVOS FL Auto cell imaging system) transfected with either Empty vector (EGFP-N1), GFP-Lamin A or Lamin B2-GFP. Across replicates, the efficiency of transfection is ~60-65%.

B) Representative mid-optical sections from confocal z-stacks of DLD-1 cells transfected with either GFP-Lamin A or Lamin B2-GFP on softer (2 kPa) matrices and glass. The Lamin constructs localize predominantly towards the nuclear periphery.

C-D) Normalized average fluorescence intensity from line-scans across nuclei for overexpressed GFP-Lamin A (C) and Lamin B2-GFP (D) in DLD-1 cells, followed by exposing them to either 2 kPa matrix or glass coverslips for 90 min. Average fluorescence intensities normalized to their respective fluorescence intensities at the nuclear periphery in cells on glass (indicated by red dot) *(Pooled data from N=2 independent biological replicates, n: number of nuclei)*. The overexpressed Lamin A and B2 show enhanced localization at the nuclear periphery (compared to the nucleoplasm) on the softer (2 kPa) matrices.

E) Dot scatter plot with median (M) % R.D for CT18 on softer matrices with overexpression of Empty vector (EGFP-N1), GFP-Lamin A, GFP-Lamin A Δ425-553, Lamin B2-GFP and Lamin B2-GFP Δ570-582 across 3 independent biological replicates after 90 mins *(n: number of each CT, 0% - Nuclear center and 100% - Nuclear periphery, LA: Lamin A, LB2: Lamin B2).*

F) Dot scatter plot with median (M) % R.D for CT19 on softer matrices with overexpression of Empty vector (EGFP-N1), GFP-Lamin A, GFP-Lamin A Δ425-553, Lamin B2-GFP and Lamin B2-GFP Δ570-582 across 3 independent biological replicates after 90 mins *(n: number of each CT, 0% - Nuclear center and 100% - Nuclear periphery, LA: Lamin A, LB2: Lamin B2).*

G) Representative western blots *(N=4)* for Lamin A, B1, B2, emerin, SUN1, SUN2 and phospho-tyrosine in DLD-1 cells with over-expression of EGFP-N1, GFP-Lamin A, Lamin B2-GFP and exposed to 2 kPa matrix for 90 mins. GAPDH was used as loading control.

H) Densitometric quantification of expression levels of Lamins, emerin and SUN proteins upon Lamin A or B2 overexpression. Expression levels were normalized to GAPDH and re-normalized to EGFP-N1 *(Error bars: SEM, N=4 independent biological replicates, Student’s t-test).*

**Figure S8. Radial positions of CT18 and CT19 are unaltered in cells overexpressing Lamins A or B2 and exposed to glass for 90 minutes**

A) Experimental scheme.

B) Representative western blots *(N=2)* for Lamin A, B1, B2, Emerin, SUN1 and SUN2 levels in DLD-1 cells upon over-expression of EGFP-N1, GFP-Lamin A, Lamin B2-GFP and exposed to glass coverslips for 90 mins. GAPDH was used as loading control.

C) Representative mid-optical sections from 3D-FISH hybridization for CT18 and 19 in DLD-1 cells on glass after overexpression of EGFP-N1, GFP-Lamin A, Lamin B2-GFP, GFP-Lamin A Δ425-553 and Lamin B2-GFP Δ570-582. 3D: reconstruction of a single representative nucleus

D) Radial distance distribution profiles for CT18 on glass after over-expression of EGFP-N1 (M=66.45%), GFP-Lamin A (M=67.39%), Lamin B2-GFP (M=66.84%), GFP-Lamin A Δ425-553 (M=65.44%) and Lamin B2-GFP Δ570-582 (M=66.26%).

E) Radial distance distribution profiles for CT19 on glass after over-expression of EGFP-N1 (M=55.32%), GFP-Lamin A (M=54.50%), Lamin B2-GFP (M=54.80%), GFP-Lamin A Δ425-553 (M=56.44%) and Lamin B2-GFP Δ570-582 (M=53.94%) *(D-E: Pooled data from N=2 independent biological replicates, n: number of CTs, X-axis: 0% - Nuclear center and 100% - Nuclear periphery, Error bar: SEM, Mann Whitney test)*. Lamin A or B2 (WT or mutant) overexpression does not perturb CT18 and CT19 positions in cells on glass.

F) Dot scatter plot with median (M) % R.D for CT18 on glass with overexpression of Empty vector (EGFP-N1), GFP-Lamin A, GFP-Lamin A Δ425-553, Lamin B2-GFP and Lamin B2-GFP Δ570-582 across 2 independent biological replicates after 90 mins *(n: number of each CT, 0% - Nuclear center and 100% - Nuclear periphery).*

G) Dot scatter plot with median (M) % R.D for CT19 on glass with overexpression of Empty vector (EGFP-N1), GFP-Lamin A, GFP-Lamin A Δ425-553, Lamin B2-GFP and Lamin B2-GFP Δ570-582 across 2 independent biological replicates after 90 mins *(n: number of each CT, 0% - Nuclear center and 100% - Nuclear periphery).* Scale bar ~10 µm.

**Figure S9. Phospho-deficient mutants of emerin show sub-cellular localization similar to wild type emerin**

A) Representative confocal (100X) and STED images showing sub-cellular localization of GFP-tagged over-expressed phospho-deficient emerin mutants (Y74F, Y95F, Y74/95FF and Y99F) and GFP-tagged over-expressed wild type (WT) emerin in DLD-1 cells on glass, counterstained for Lamin B1.

B) Representative confocal (100X) images showing sub-cellular localization of GFP-tagged over-expressed phospho-deficient emerin mutants (Y74F, Y95F, Y74/95FF and Y99F) and GFP-tagged over-expressed wild type (WT) emerin in DLD-1 cells on 2 kPa matrices, counterstained for Lamin B1.

C) Representative confocal (63X) images showing sub-cellular localization of GFP-tagged over-expressed phospho-deficient emerin mutants (Y74F, Y95F, Y74/95FF and Y99F) and GFP-tagged over-expressed wild type (WT) emerin in DLD-1 cells on glass, counterstained for Lamin B1 and DAPI. Scale bar ~10 µm.

**Figure S10. Inhibition of Emerin phosphorylation selectively abrogates chromosome territory movements**

A) Dot scatter plot with median (M) % R.D for CT18 on softer (2 kPa) matrices and glass, with and without (DMSO control) PP2 treatment for 90 mins across 2 independent biological replicates after 90 mins *(n: number of each CT, 0% - Nuclear center and 100% - Nuclear periphery).*

B) Dot scatter plot with median (M) % R.D for CT19 on softer (2 kPa) matrices and glass, with and without (DMSO control) PP2 treatment for 90 mins across 2 independent biological replicates after 90 mins *(n: number of each CT, 0% - Nuclear center and 100% - Nuclear periphery).*

C) Dot scatter plot with median (M) % R.D for CT1 on softer (2 kPa) matrices and glass, with and without (DMSO control) PP2 treatment for 90 mins across 2 independent biological replicates *(n: number of each CT, 0% - Nuclear center and 100% - Nuclear periphery).*

D) Dot scatter plot with median (M) % R.D for CT18 (co-labelled with CT1) on softer (2 kPa) matrices and glass, with and without (DMSO control) PP2 treatment for 90 mins across 2 independent biological replicates *(n: number of each CT, 0% - Nuclear center and 100% - Nuclear periphery).*

**Overexpression of Emerin Y99F in cells on softer (2 kPa) matrices enhances localization of Lamin A and B2 at the nuclear periphery**

E) Representative mid-optical sections of (DLD-1) empty vector and shEmerin clones immunostained for emerin. shEmerin clone shows pronounced downregulation of emerin as compared to the empty vector clone.

F) Representative mid-optical sections *(N=2 independent biological replicates)* from confocal z-stacks of Lamin A and B2 immunostaining in vector control and shEmerin clones on 2 kPa matrices overexpressing EGFPN1, GFP-Emerin WT or GFP-Emerin Y99F.

G-H) Normalized average fluorescence intensity from line-scans across nuclei performed for Lamin A and B2 *(images from F)* in vector control and shEmerin clones on 2 kPa matrices overexpressing EGFPN1, GFP-Emerin WT or GFP-Emerin Y99F *(F-H, EMD: emerin,* *Pooled data from N=2 independent biological replicates, n: number of nuclei, Error bar: SEM, Student’s t-test)*. ** p<0.01, * p<0.05. Scale bar ~10 µm.

**Figure S11. Overexpression of Emerin Y99F selectively abrogates mislocalization of chromosome territories**

A) Experimental scheme.

B) Representative mid-optical sections from 3D-FISH hybridizations for CT18 and 19 in vector control clones on 2 kPa matrix after overexpression of Empty vector (EGFP-N1), WT Emerin (GFP-EMD WT) and Emerin Y99F (GFP-EMD Y99F). Arrowheads show specific hybridization for CT18 and CT19, resolved in 3D: reconstruction of single representative nucleus.

C) Radial distance distribution profiles for CT18 in vector control clone (on 2 kPa) after over-expression of Empty vector (*N=3*, M=51.43%), GFP-EMD WT (*N=3*, M=54.59%) and GFP-EMD Y99F (*N=3*, M=56.22%).

D) Radial distance distribution profiles for CT19 in vector control clone (on 2 kPa) after over-expression of Empty vector (*N=3*, M=42.74%), GFP-EMD WT (*N=3*, M=43.69%) and GFP-EMD Y99F (*N=3*, M=47.49%) *(B-D, EMD: emerin,* *Pooled data from N=3 independent biological replicates, n: number of CTs, X-axis: 0% - Nuclear center and 100% - Nuclear periphery, Error bar: SEM)*.

E) Dot scatter plot with median (M) % R.D for CT18 in vector control and shEmerin clones on 2 kPa matrix after over-expression of EGFPN1, GFP-EMD WT and GFP-EMD Y99F across 3 independent biological replicates *(n: number of each CT, 0% - Nuclear center and 100% - Nuclear periphery).*

F) Dot scatter plot with median (M) % R.D for CT19 in vector control and shEmerin clones on 2 kPa matrix after over-expression of EGFPN1, GFP-EMD WT and GFP-EMD Y99F across 3 independent biological replicates *(E-F, EMD: emerin, n: number of each CT, 0% - Nuclear center and 100% - Nuclear periphery).*

G) Dot scatter plot with median (M) % R.D for CT1 in vector control and shEmerin clones on 2 kPa matrix after over-expression of EGFPN1, GFP-EMD WT and GFP-EMD Y99F across 2 independent biological replicates *(For G, EMD: emerin, n: number of each CT, 0% - Nuclear center and 100% - Nuclear periphery).* Scale bar ~10 µm.

**Figure S12.**

A-C) Selected optical sections showing hybridization of chromosome 18 (green) and 19 (red) territories in cells on 2 kPa matrix (A), 55 kPa matrix (B) and glass (C).

**Supplementary Tables**

**Table S1. Nuclear and cell surface areas for DLD1 cells on softer matrices (2 kPa and 55 kPa) across time**

| **Time (min)** | **Nuclear area (μm^2^)** | | **Cell area (μm^2^)** | |
| --- | --- | --- | --- | --- |
|  | **2 kPa** | **55 kPa** | **2 kPa** | **55 kPa** |
| 15 | 97.28 | 115.1 | 183.9 | 241.2 |
| 30 | **112.4*** | **137.9*** | **241.8*** | **318.6*** |
| 45 | 112.2 | 134.6 | 280.6 | **367.2*** |
| 60 | **133.0*** | **161.2*** | 283.5 | 381.1 |
| 90 | 137.0 | 162.1 | 336.1 | **413.9*** |
| 145 | 138.5 | 164.5 | 327.2 | 469.4 |
| 420 | 131.8 | 162.1 | 354.1 | 482.8 |
| 1260 | 130.5 | 152.7 | 335.4 | 479.0 |

*p<0.05

**Table S2. % deregulation of all chromosomes in cells on softer matrices (2 kPa and 55 kPa)**

|  | **2 kPa** | | **55 kPa** | |
| --- | --- | --- | --- | --- |
| **Chromosome** | **%Upregulation**  **(normalized to total transcribing genes with FPKM>1 on respective chromosome)** | **%Downregulation**  **(normalized to total transcribing genes with FPKM>1 on respective chromosome)** | **%Upregulation**  **(normalized to total transcribing genes with FPKM>1 on respective chromosome)** | **%Downregulation**  **(normalized to total transcribing genes with FPKM>1 on respective chromosome)** |
| 1 | 7.23 | 9.40 | 4.00 | 5.13 |
| 2 | 3.93 | 3.58 | 3.50 | 3.23 |
| 3 | 4.87 | 6.19 | 4.13 | 5.77 |
| 4 | 2.40 | 3.20 | 2.90 | 4.14 |
| 5 | 3.60 | 4.01 | 3.40 | 3.53 |
| 6 | 3.49 | 5.04 | 3.04 | 2.94 |
| 7 | 5.52 | 5.16 | 2.94 | 6.00 |
| 8 | 6.13 | 5.20 | 3.70 | 5.21 |
| 9 | 3.71 | 4.95 | 5.78 | 3.96 |
| 10 | 4.54 | 5.64 | 4.10 | 4.49 |
| 11 | 4.58 | 6.87 | 4.15 | 6.69 |
| 12 | 5.77 | 4.68 | 4.41 | 5.10 |
| 13 | 3.49 | 3.49 | 3.02 | 2.76 |
| 14 | 5.03 | 5.39 | 2.80 | 3.55 |
| 15 | 3.85 | 3.46 | 3.21 | 3.04 |
| 16 | 3.40 | 5.18 | 3.32 | 5.21 |
| 17 | 6.14 | 4.44 | 6.42 | 6.42 |
| 18 | 3.26 | 2.79 | 3.77 | 3.77 |
| 19 | 5.62 | 6.21 | 3.90 | 6.19 |
| 20 | 5.49 | 2.47 | 5.21 | 4.95 |
| 21 | 5.08 | 3.95 | 4.12 | 4.71 |
| 22 | 6.85 | 5.95 | 5.38 | 3.97 |
| X | 4.15 | 4.15 | 4.24 | 6.46 |
| Y | 7.14 | 0.00 | 0.00 | 0.00 |

**Table S3. Enrichment of deregulated genes in cells on softer matrices (2 kPa and 55 kPa) on all chromosomes**

|  | **2 kPa** | | **55 kPa** | |
| --- | --- | --- | --- | --- |
| **Chromosome** | **%Upregulated**  **(out of 783 genes)** | **%Downregulated**  **(out of 872 genes)** | **%Upregulated**  **(out of 649 genes)** | **%Downregulated**  **(out of 783 genes)** |
| 1 | 15.27 | 18.22 | 8.52 | 10.63 |
| 2 | 7.12 | 5.96 | 6.62 | 5.84 |
| 3 | 5.60 | 6.54 | 4.83 | 6.89 |
| 4 | 2.29 | 2.80 | 2.67 | 3.74 |
| 5 | 3.44 | 3.50 | 3.44 | 3.27 |
| 6 | 4.58 | 6.07 | 3.94 | 3.74 |
| 7 | 5.85 | 5.02 | 3.05 | 5.84 |
| 8 | 4.20 | 3.27 | 2.80 | 3.74 |
| 9 | 3.05 | 3.74 | 4.83 | 3.15 |
| 10 | 4.20 | 4.79 | 3.94 | 4.44 |
| 11 | 4.83 | 6.66 | 4.58 | 6.89 |
| 12 | 6.74 | 5.02 | 4.83 | 5.14 |
| 13 | 1.78 | 1.64 | 1.53 | 1.40 |
| 14 | 3.56 | 3.50 | 1.91 | 2.34 |
| 15 | 2.54 | 2.10 | 2.29 | 2.10 |
| 16 | 2.67 | 3.74 | 2.67 | 4.32 |
| 17 | 5.98 | 3.97 | 6.49 | 6.31 |
| 18 | 0.89 | 0.70 | 1.02 | 0.93 |
| 19 | 6.11 | 6.19 | 4.33 | 6.66 |
| 20 | 2.54 | 1.05 | 2.54 | 2.34 |
| 21 | 1.15 | 0.82 | 0.89 | 0.93 |
| 22 | 2.93 | 2.34 | 2.42 | 2.10 |
| X | 2.54 | 2.34 | 2.67 | 3.97 |
| Y | 0.13 | 0.00 | 0.00 | 0.00 |

**Table S4. Radial distance measurements for CT18 and CT19 under conditions of altered extracellular matrix stiffness across replicates**

| **Substrate/**  **Conditions** | **Median % Radial Distance (% R.D)** | | | | | |
| --- | --- | --- | --- | --- | --- | --- |
|  | **CT18** | | | **CT19** | | |
|  | N=1 | N=2 | N=3 | N=1 | N=2 | N=3 |
| 2 kPa (90 min) | 58.06 | 53.00 | 58.67 | 49.97 | 48.92 | 48.51 |
| 55 kPa (90 min) | 60.41 | 58.40 | 60.01 | 53.27 | 47.52 | 48.58 |
| Glass (90 min) | 66.32 | 65.98 | 66.58 | 55.92 | 50.68 | 56.04 |
| 2 kPa to glass | 68.69 | 66.51 | 66.77 | 43.55 | 48.82 | 46.40 |
| 55 kPa to glass | 73.09 | 67.72 | 66.02 | 52.79 | 49.85 | 57.33 |
| EGFP-N1 on 2 kPa | 59.18 | 56.79 | 51.01 | 51.86 | 52.32 | 46.21 |
| GFP-Lamin A on 2 kPa | 45.77 | 47.25 | 46.74 | 58.09 | 59.10 | 57.63 |
| Lamin B2-GFP on 2 kPa | 66.63 | 69.56 | 65.98 | 54.29 | 53.51 | 52.43 |
| GFP-Lamin A Δ425-553 on 2 kPa | 58.80 | 58.41 | 51.13 | 47.92 | 51.26 | 46.61 |
| Lamin B2-GFP Δ570-582 on 2 kPa | 56.20 | 54.23 | 51.80 | 49.40 | 49.19 | 46.90 |
| EGFP-N1 on Glass | 66.16 | 66.89 | - | 53.91 | 55.75 | - |
| GFP-Lamin A on 2 kPa | 67.35 | 67.39 | - | 55.84 | 50.98 | - |
| Lamin B2-GFP on Glass | 66.50 | 67.18 | - | 53.79 | 55.03 | - |
| GFP-Lamin A Δ425-553 on Glass | 64.81 | 66.06 | - | 56.83 | 54.95 | - |
| Lamin B2-GFP Δ570-582 on Glass | 67.84 | 64.86 | - | 52.39 | 55.11 | - |
| Glass + DMSO | 66.09 | 66.32 | - | 51.01 | 55.91 | - |
| Glass + 20 µM PP2 | 68.02 | 67.78 | - | 54.60 | 54.51 | - |
| 2 kPa + DMSO | 55.79 | 52.64 | - | 48.80 | 49.19 | - |
| 2 kPa + 20 µM PP2 | 67.97 | 66.38 | - | 53.87 | 57.72 | - |
| Vector control + EGFP-N1 on 2 kPa | 53.76 | 48.12 | 51.64 | 42.01 | 44.80 | 41.65 |
| Vector control + WT-EMD on 2 kPa | 52.56 | 55.99 | 53.59 | 43.53 | 46.20 | 41.09 |
| Vector control + EMD Y99F on 2 kPa | 58.40 | 59.12 | 50.33 | 48.16 | 49.88 | 42.76 |
| shEmerin + EGFP-N1 on 2 kPa | 62.16 | 61.81 | 61.26 | 51.14 | 50.93 | 51.37 |
| shEmerin + WT-EMD on 2 kPa | 56.10 | 57.41 | 51.02 | 47.00 | 50.69 | 42.22 |
| shEmerin + EMD Y99F on 2 kPa | 65.45 | 64.67 | 67.90 | 53.16 | 52.92 | 54.84 |

N: indicates independent biological replicates.

**Table S5. Radial distance measurements for CT1 under conditions of altered extracellular matrix stiffness across replicates**

| **Substrate/**  **Conditions** | **Median % Radial Distance (% R.D)** | |
| --- | --- | --- |
|  | **CT1** | |
|  | N=1 | N=2 |
| 2 kPa (90 min) | 48.26 | 51.21 |
| Glass (90 min) | 68.16 | 66.28 |
| Glass + DMSO | 59.73 | 64.00 |
| Glass + 20 µM PP2 | 57.02 | 63.00 |
| 2 kPa + DMSO | 48.48 | 51.53 |
| 2 kPa + 20 µM PP2 | 51.50 | 47.76 |
| Vector control + EGFP-N1 on 2 kPa | 46.16 | 45.43 |
| shEmerin + EGFP-N1 on 2 kPa | 39.63 | 35.64 |
| shEmerin + WT-EMD on 2 kPa | 45.80 | 50.37 |
| shEmerin + EMD Y99F on 2 kPa | 43.15 | 44.29 |

N: indicates independent biological replicates.
