# Supplementary figures and images for: Emerin modulates spatial organization of chromosome territories in cells on softer matrices
Source: Nucleic Acids Res. 2018 Apr 19;46(11):5561–86. doi: 10.1093/nar/gky288 (PMC6009696; doi:10.1093/nar/gky288)

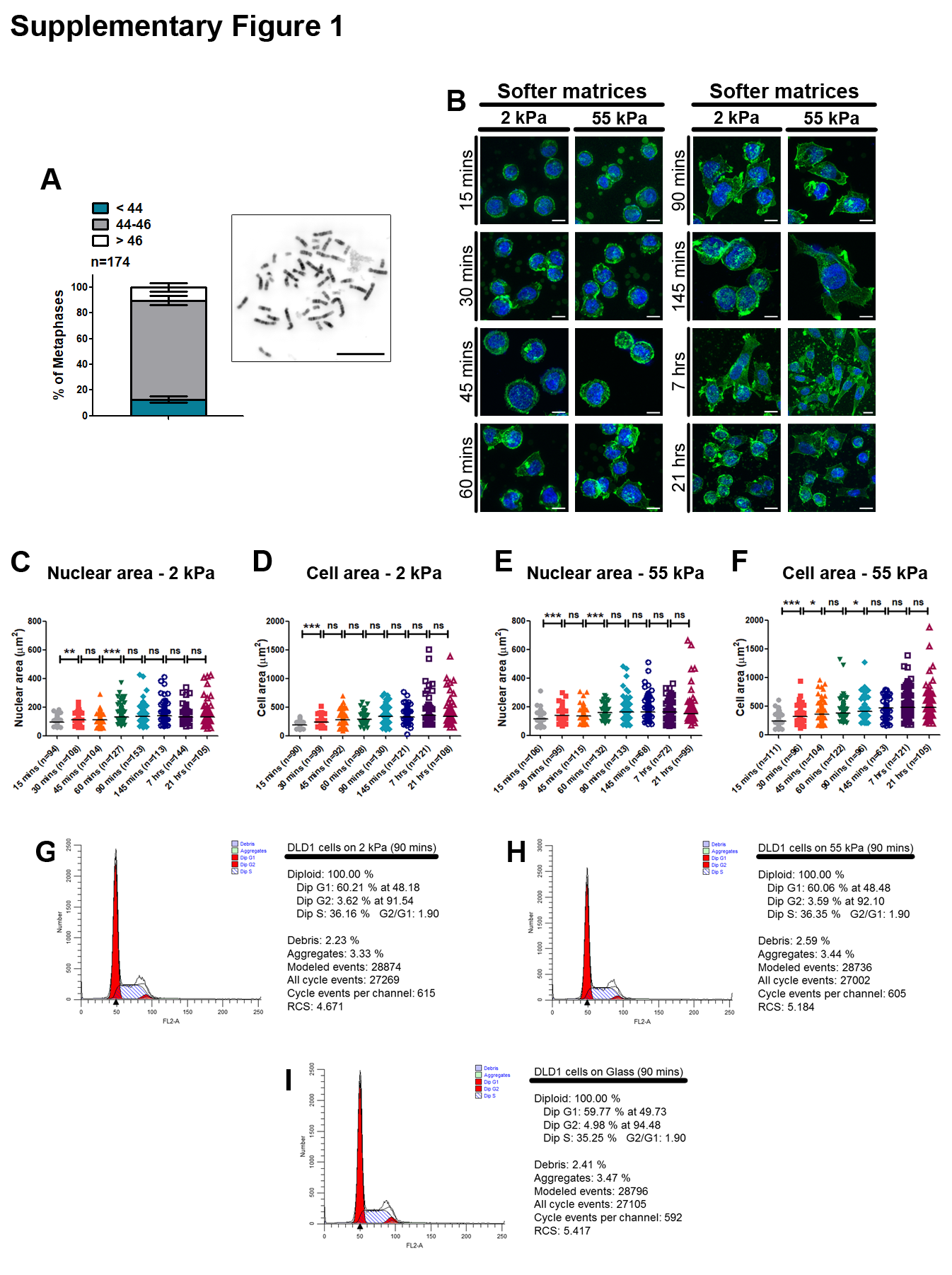

Supplement: Supplementary Data [file gky288_supplemental_files.zip › Suppl Fig 1.tif]

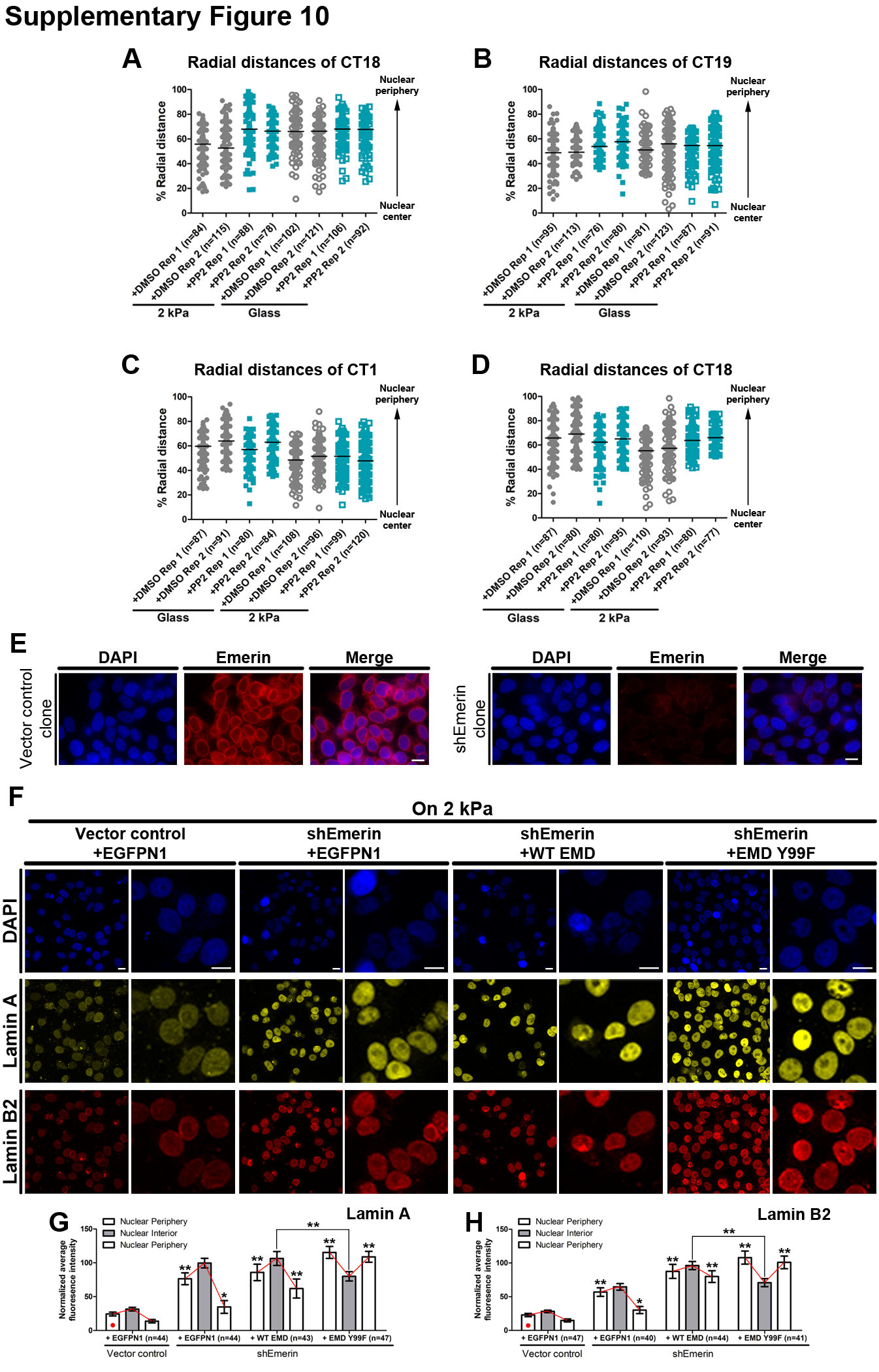

Supplement: Supplementary Data [file gky288_supplemental_files.zip › Suppl Fig 10.tif]

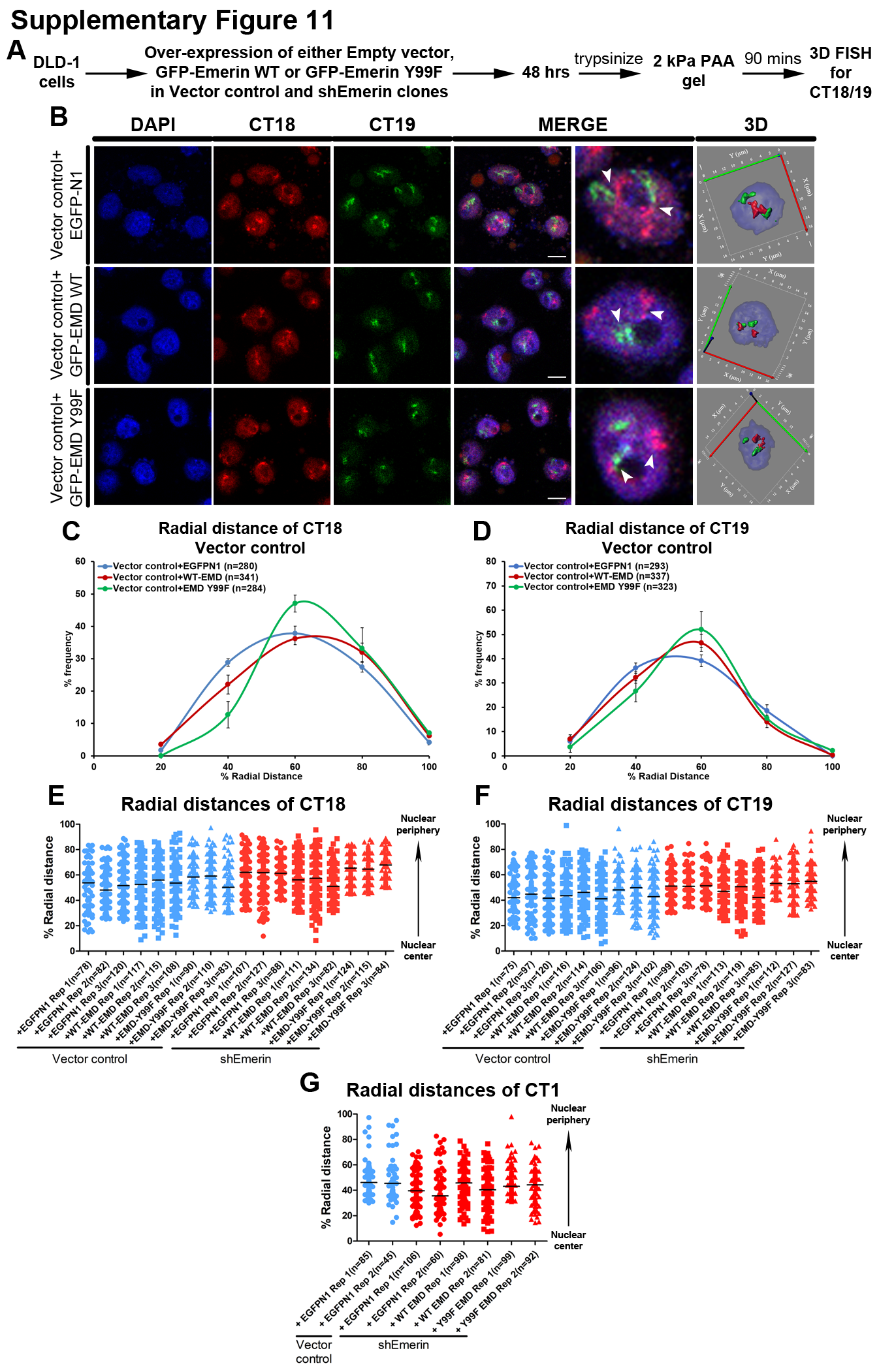

Supplement: Supplementary Data [file gky288_supplemental_files.zip › Suppl Fig 11.tif]

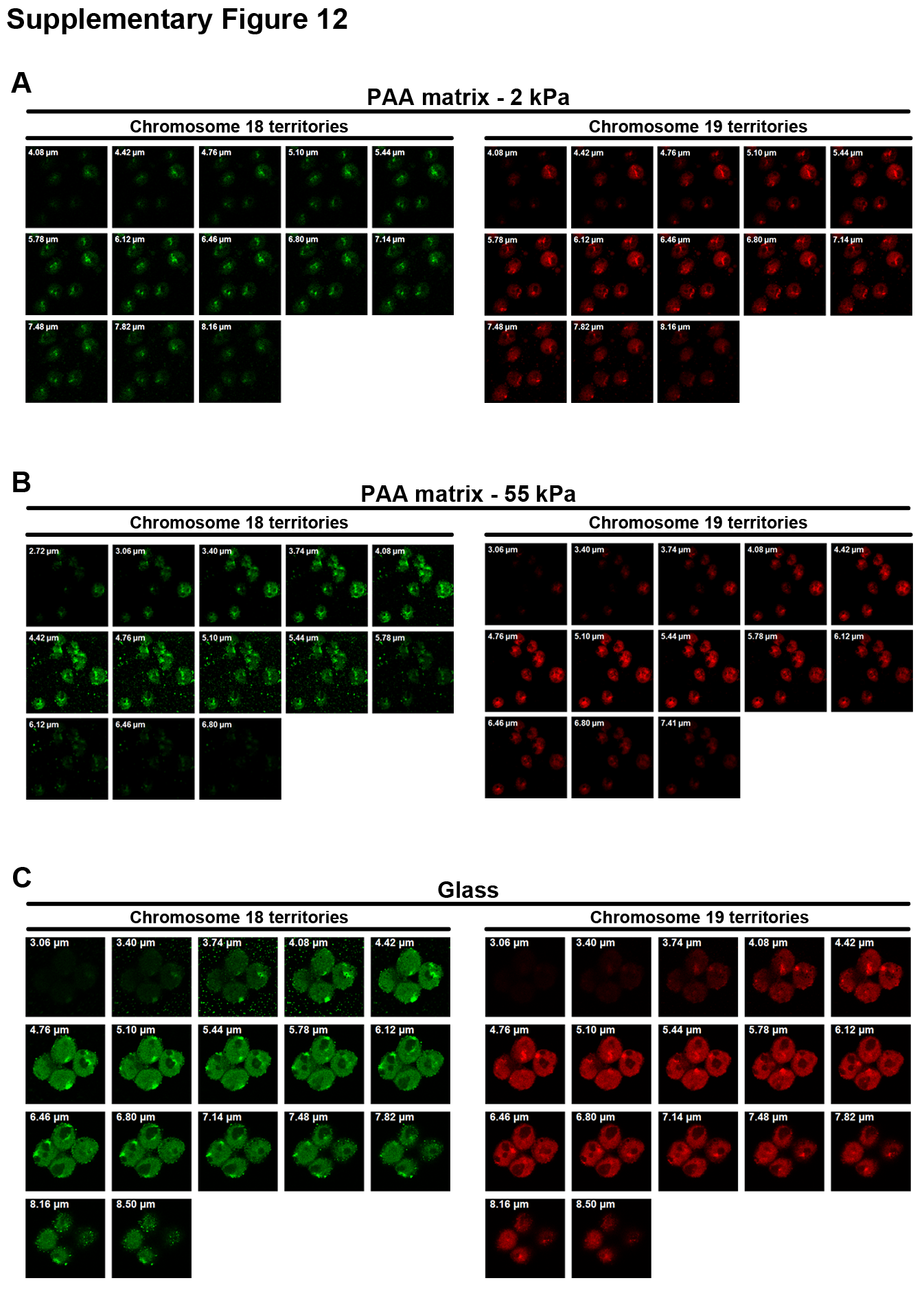

Supplement: Supplementary Data [file gky288_supplemental_files.zip › Suppl Fig 12.tif]

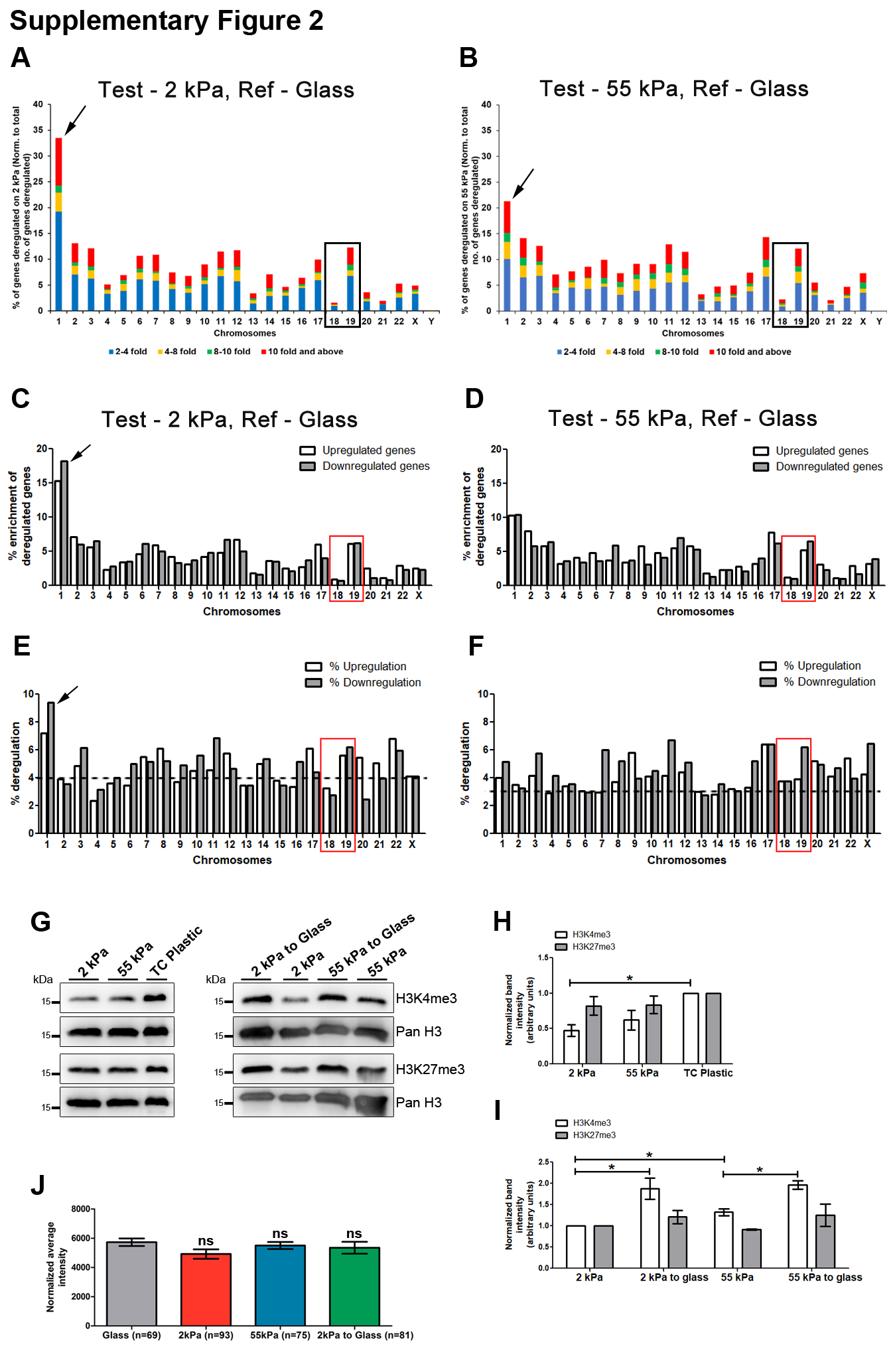

Supplement: Supplementary Data [file gky288_supplemental_files.zip › Suppl Fig 2.tif]

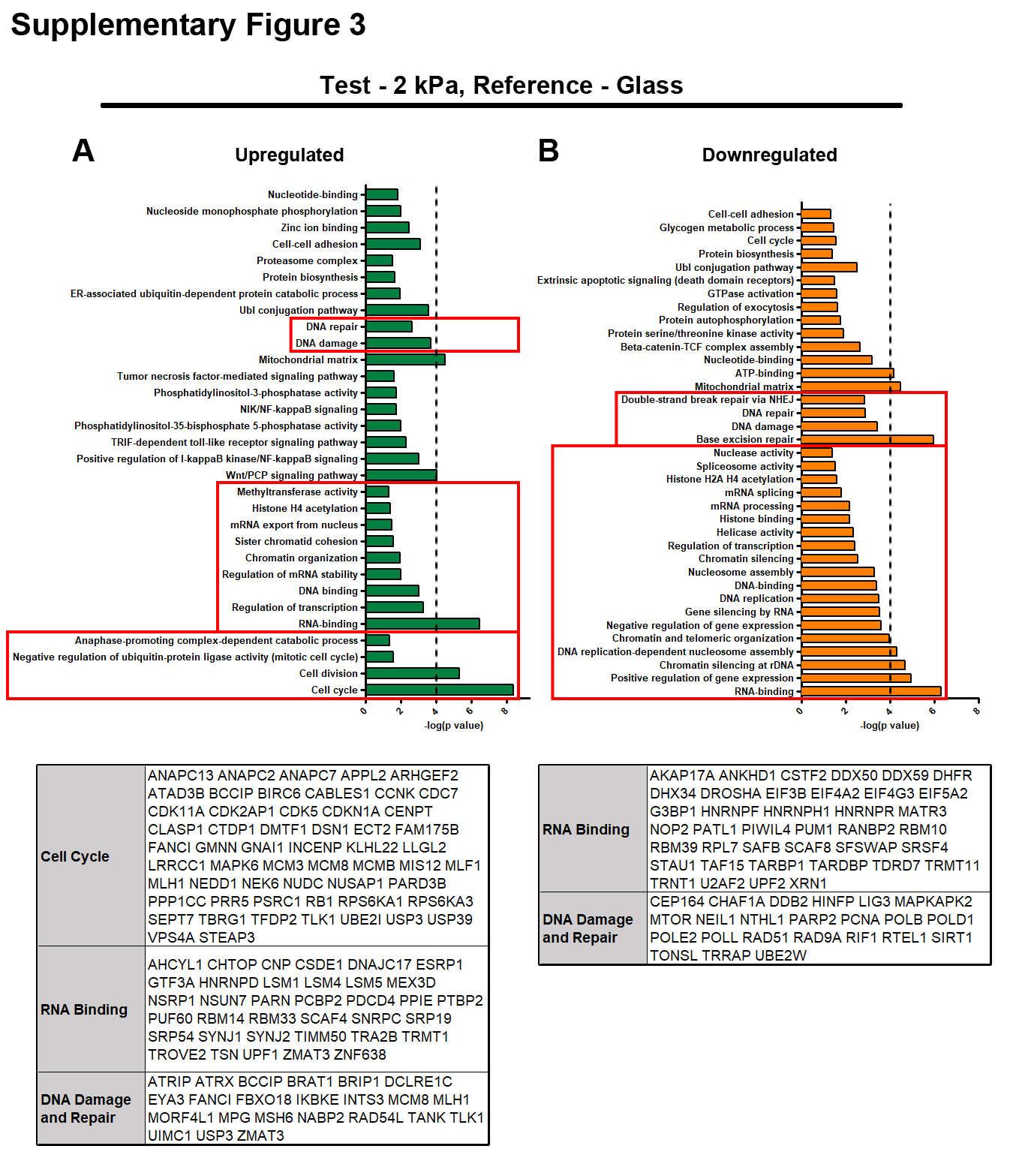

Supplement: Supplementary Data [file gky288_supplemental_files.zip › Suppl Fig 3.tif]

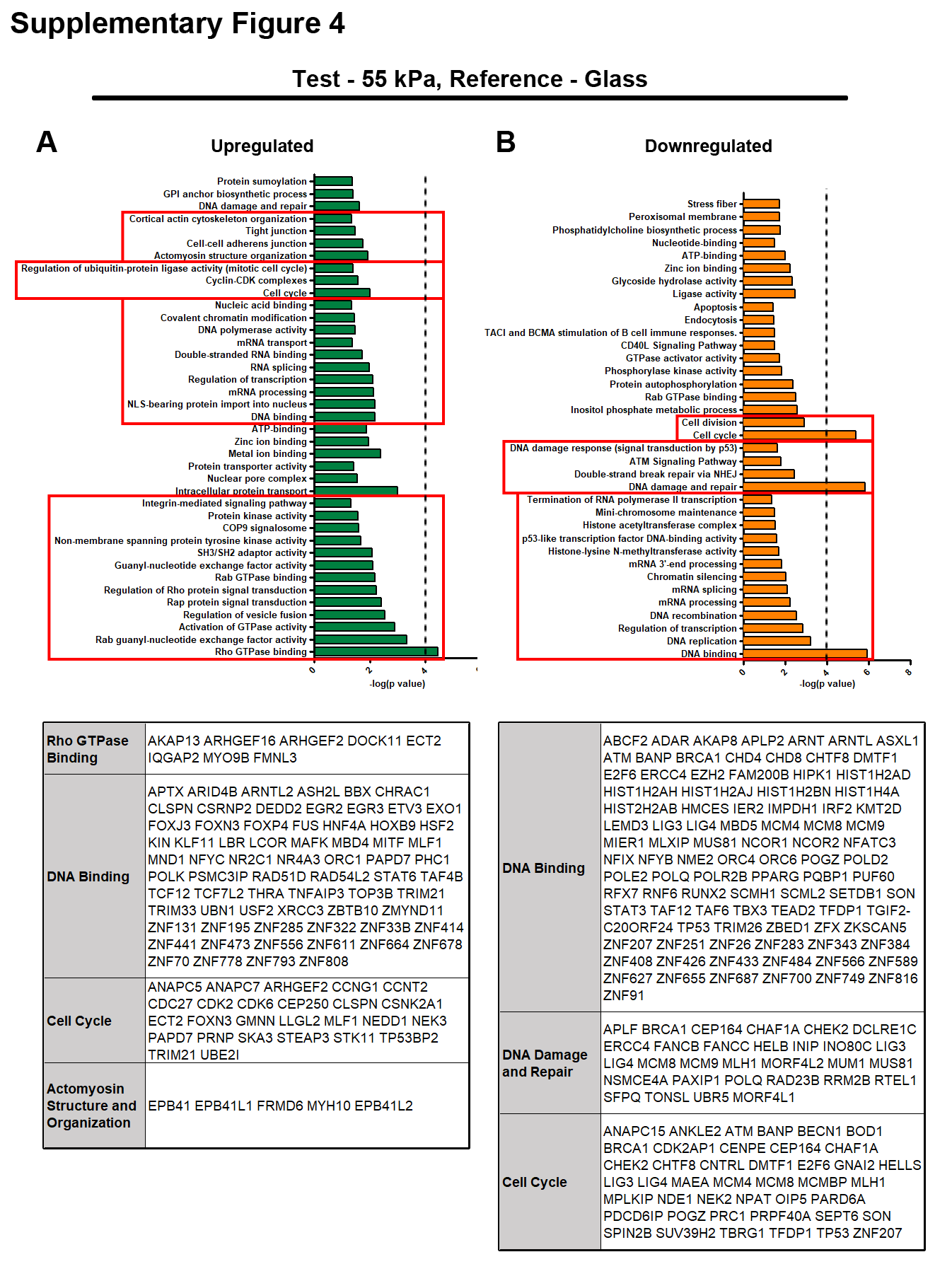

Supplement: Supplementary Data [file gky288_supplemental_files.zip › Suppl Fig 4.tif]

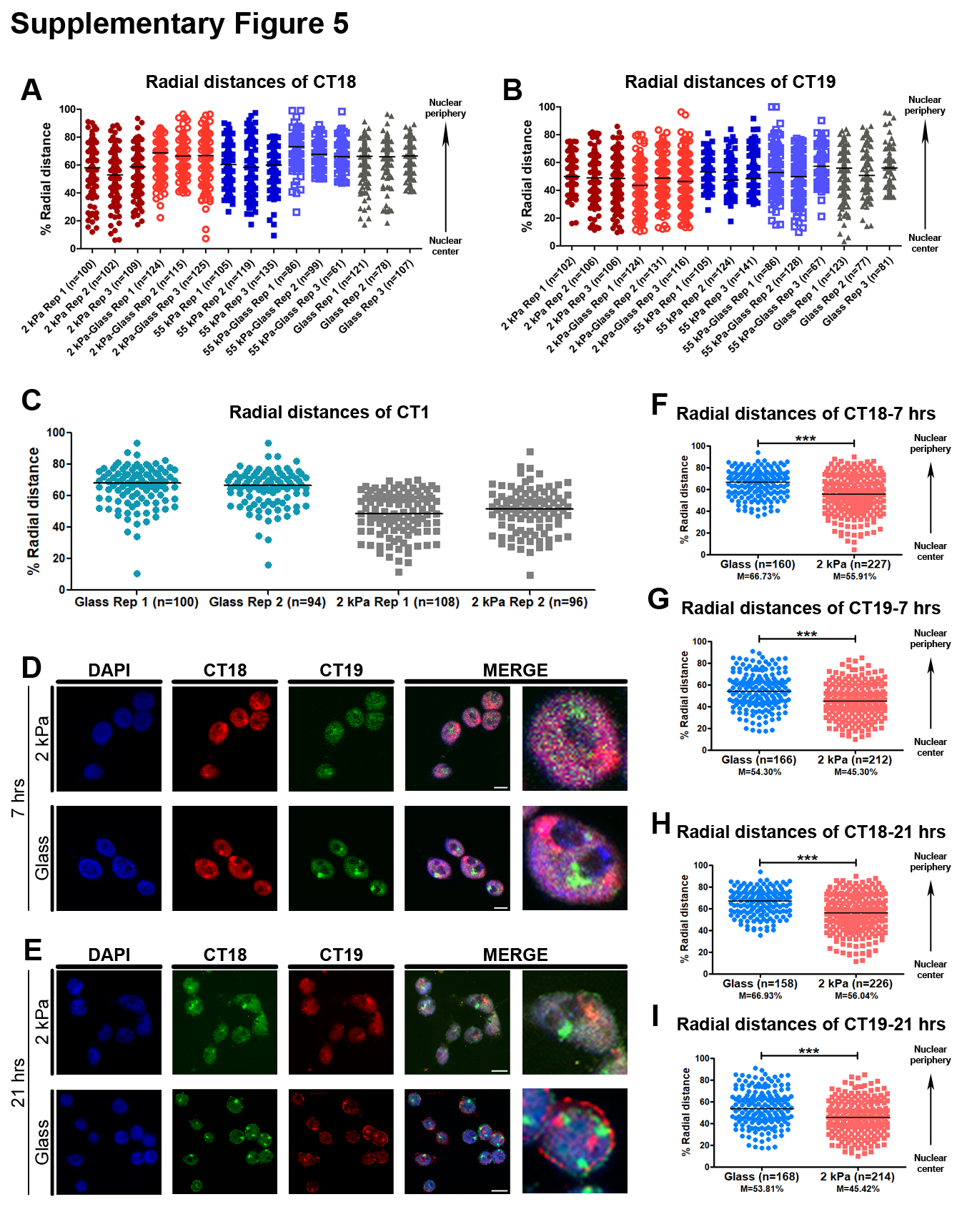

Supplement: Supplementary Data [file gky288_supplemental_files.zip › Suppl Fig 5.tif]

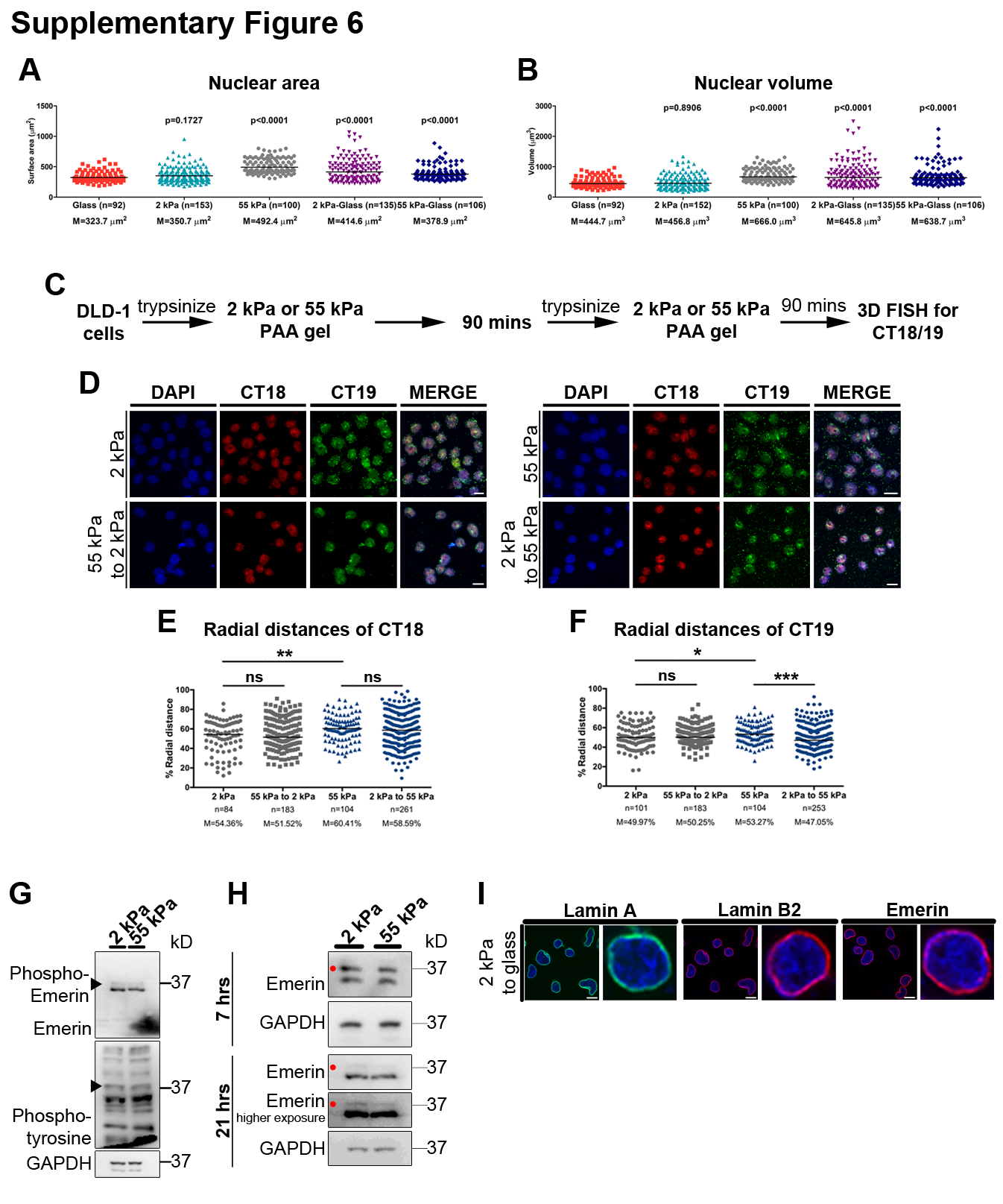

Supplement: Supplementary Data [file gky288_supplemental_files.zip › Suppl Fig 6.tif]

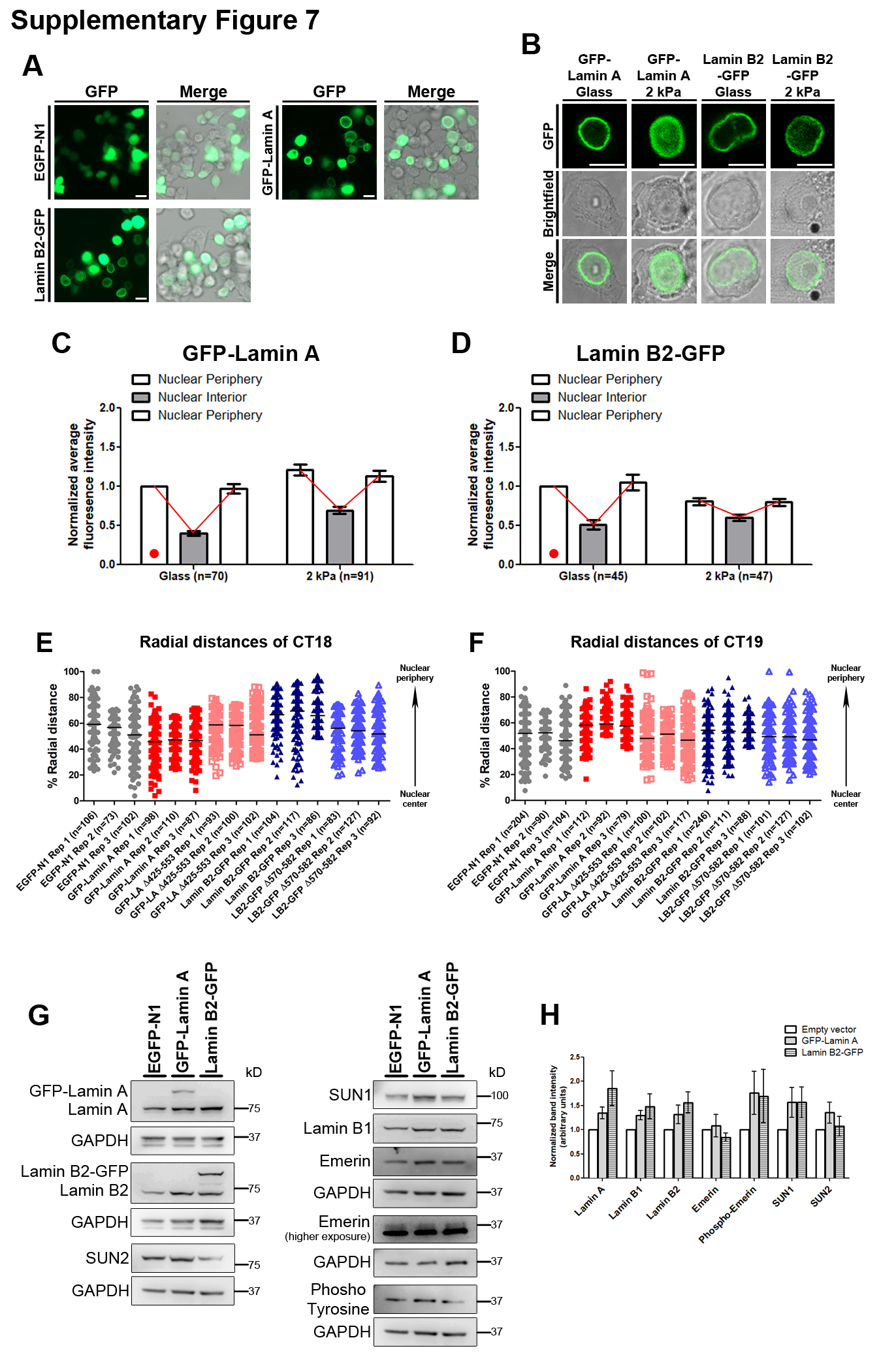

Supplement: Supplementary Data [file gky288_supplemental_files.zip › Suppl Fig 7.tif]

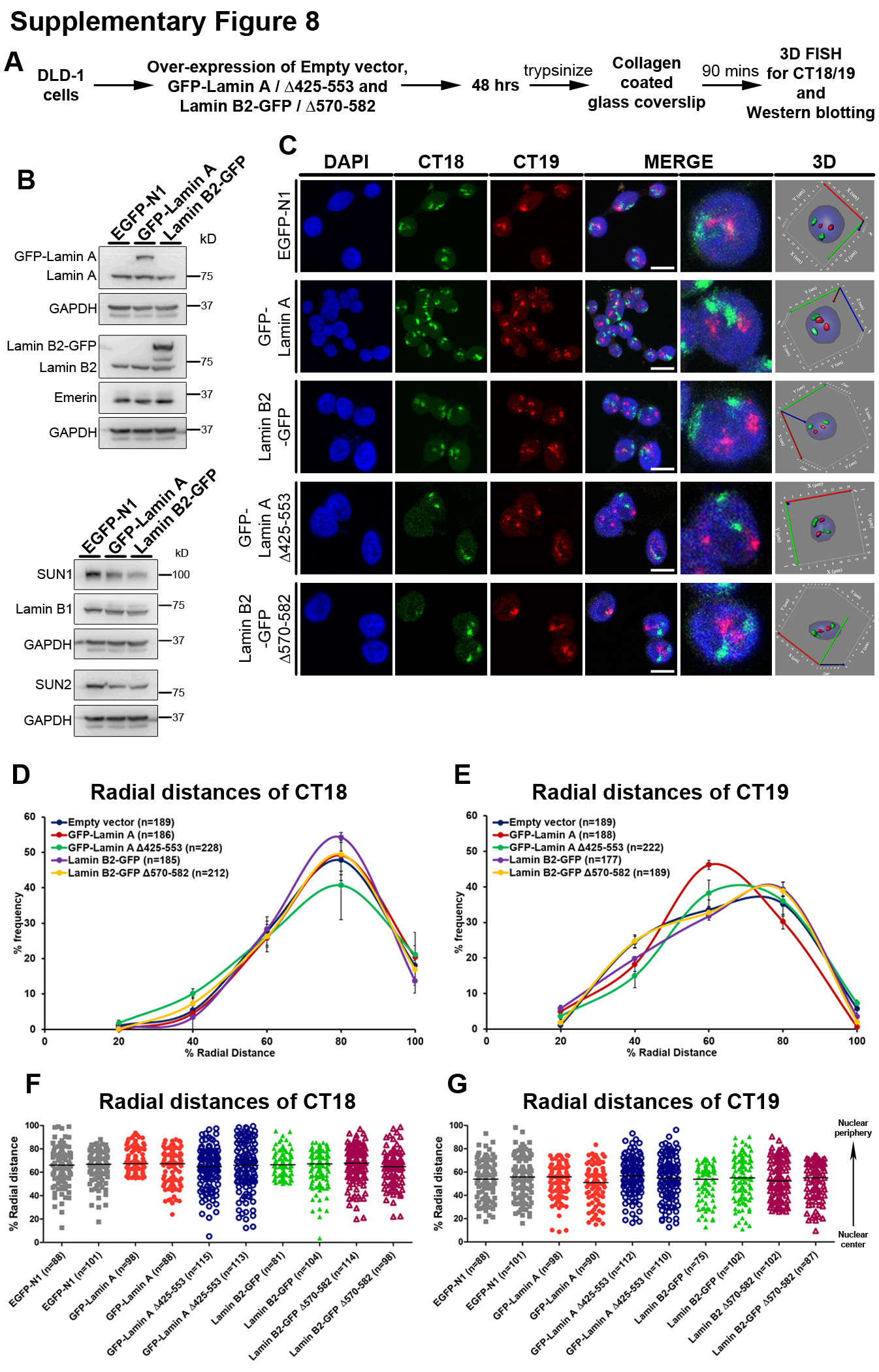

Supplement: Supplementary Data [file gky288_supplemental_files.zip › Suppl Fig 8.tif]

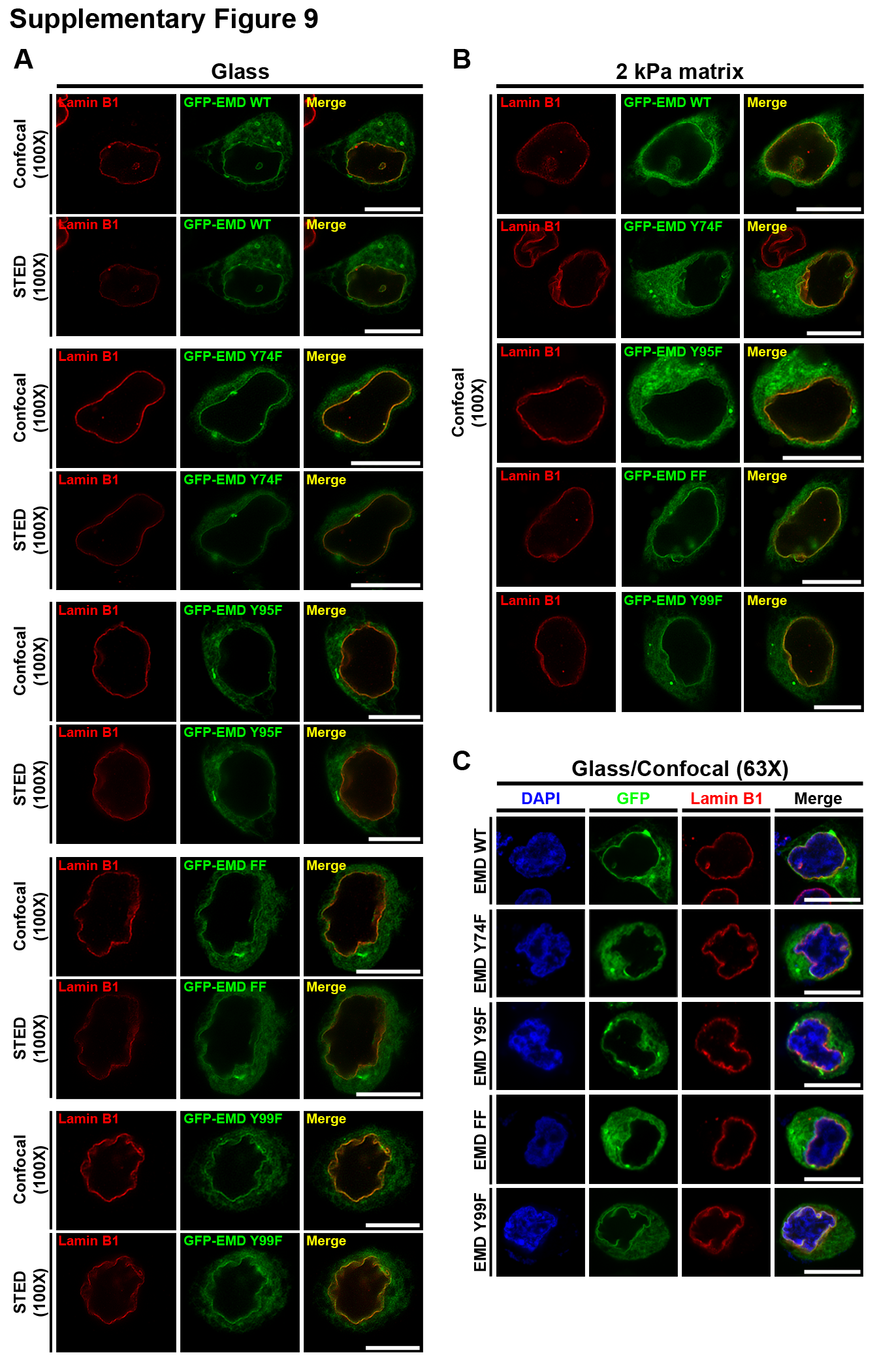

Supplement: Supplementary Data [file gky288_supplemental_files.zip › Suppl Fig 9.tif]
